# Supplementary material for: Cell therapy for brain tumors: The first 60 years
Source: Cell Rep Med. 2026 Feb 17;7(2):102626. doi: 10.1016/j.xcrm.2026.102626 (PMC12923960; doi:10.1016/j.xcrm.2026.102626)
Supplement: Document S1. Tables S1 and S2 [file mmc1.pdf]

Cell Reports Medicine, Volume *7*

Supplemental information

Cell therapy for brain tumors: The first 60 years

Sanya Mehta, Giedre Krenciute, and Stephen Gottschalk

Supplementary Table 1: Cell Therapy with lymphokine-activated killer cells

Year(s)	Pt(#)	Diagnosis	Treatment	Cell dose	Best clinical outcomes
1986[15,50]	10	Progressive glioma	auto LAKs and/or IL-2	$0.08\text{-}1 \times 10^{10}$ (ITu)	No benefit
1987[54]	1	Meningeal gliomatosis	auto LAKs and IL-2	$1\text{-}2 \times 10^{8}$ (ITu)	Improvement in symptoms for 7 mths
1987[55] 1988[58,59]	23	Recurrent anaplastic A	auto LAKs and IL-2	$1.2\text{-}3.24 \times 10^{8}$ (ITu)	1 pt: CR for >14 mths<br>1 pt: partial regression with no recurrence for >19 mths<br>2 pts: transient regression for 6-8 mths<br>7 pts: improvement in symptoms
1988[81] 1989[63]	6	MB with CSF dissemination	haplo LAKs and IL-2	$0.2\text{-}1 \times 10^{9}$ (ITu or IT)	1 pt: CR for >18 mths<br>3 pts: improvement in
1988[56] 1989[60]	13	Recurrent GBM	auto LAKs and IL-2	$0.1\text{-}5.8 \times 10^{9}$ (ITu)	1 pt: no recurrence for >12 mths
1988[57] 1989[60]	19	Primary or recurrent GBM or high-grade OG	auto LAKs and IL-2 during craniotomy	$0.1\text{-}1.5 \times 10^{10}$ (ITu)	8 pts: progression free for >6 mths
1989[61]	9	Progressive GBM and anaplastic A	auto LAKs and IL-2	$0.09\text{-}2.1 \times 10^{10}$ (ITu)	1 pt: PR for 9 mths, then SD for 4 mths
1989[62] 1997[76]	1	Recurrent anaplastic glioma	auto LAKs with IFN-β and ANCU	2.7×10^{9} (ITu)	CR for >9 years and 9 months
1990[65,66]	20	Primary GBM or anaplastic A	auto LAKs +/- bispecific antibody	$0.8\text{-}1.2 \times 10^{8}$ (ITu)	4 pts: CR for >8-18 mths<br>5 pts: partial regression for >8-18 mths
1990[64] 1994[73]	9	Recurrent GBM or anaplastic A	adherent auto LAKs and IL-2	1.5×10^{8} (ITu)	1 pt: CR for 28 wks<br>2 pts: PR for 25-28 wks<br>4 pts: SD for 12-36 wks
1991[67]	20	Recurrent GBM or anaplastic A	auto LAKs, stimulated lymphocytes, and IL-2	$0.19\text{-}2.75 \times 10^{10}$ (ITu)	No benefit
1992[68]	1	GBM or grade 3 A	auto LAKs and IL-2 during craniotomy, PHA-stimulated and tumor-sensitized lymphocytes	Unknown (ITu)	No benefit
1993[71]	19	Recurrent grade 3-4 HGG	auto LAKs and IL-2 during craniotomy +/- PHA coating of surgical cavity	$0.22\text{-}5.8 \times 10^{10}$ (ITu)	3 pts: transient regression for <4 mths<br>7 pts: Improvement in clinical symptoms for <4-8 mths
1993[72]	1	Recurrent grade 3 A	auto LAKs and IL-2	Unknown (ITu)	No benefit
1993[70]	8	Progressive GBM and anaplastic A	auto LAKs and IL-2	$0.55\text{-}9.37 \times 10^{8}$ (ITu)	1 pt: CR for >5 yrs<br>2 pt: PR for 6 and 11 mths
1993[69]	5	Recurrent GBM	auto LAKs and IL-2	Unknown (ITu)	No benefit
1994[51]	1	MB with CSF dissemination	auto LAKs and IL-2	7×10^{8} (IT)	CR for >30 mths
1995[74]	9	Primary or recurrent GBM or grade 3 A	auto LAKs and IL-2 +/- XRT	$3.7\text{-}9.49 \times 10^{7}$ (ITu)	2 pts: 2 CR for >20 and >90 mths<br>1 pt: PR for >24 mths<br>1 pt: transient PR
1995[53] 2001[77,78]	28	Primary or recurrent malignant glioma	auto LAKs and IL-2	$0.5\text{-}5 \times 10^{9}$ (ITu)	2 pts: CR for >3.4 yrs<br>1 pt: CR for >27 mths<br>2 pts: PR for 9.5 mths and 3.6 yrs<br>1 pt: SD for >3 yrs
1996[75]	10	Recurrent grade 2-3 A, GBM, AO, MB	auto LAKs	$1\text{-}2.5 \times 10^{6}$ (ITu)	2 pts: neurological improvements for 18 and 56 wks
2004[79]	40	Recurrent GBM	auto LAKs during craniotomy	$1\text{-}3 \times 10^{9}$ (ITu)	Improved survival compared to historical ctrls (avg: 17.5 vs 13.6 mths)
2009[80]	33	Primary GBM or grade 3 A following std primary therapy	auto LAKs during craniotomy	$0.93\text{-}2.57 \times 10^{9}$ (ITu)	Improved survival compared to historical ctrls (avg: 20.5 vs 12 mths)

Pt(#): Number of patients, ***Diagnosis column:*** A: astrocytoma, AO: astrocytoma-oligodendroglioma, CSF: cerebrospinal fluid, GBM: glioblastoma, HGG: high-grade glioma, MB: medulloblastoma, MM: metastatic meningioma, OA: oligoastrocytoma, OG: oligodendroglioma, std: standard*; **Treatment column:*** allo: allogeneic, ANCU: 1-(4-amino-2-methyl-5-pyrimidinyl) methyl-3-(2- chloroethyl)-3-nitrosourea hydrochloride, auto: autologous, haplo: haploidentical, IFN: interferon, IL: interleukin, lymphokine-activated killer cells: LAKs, PBMCs: peripheral blood mononuclear cells, PHA: phytohemagglutinin, XRT: radiation therapy*; **Cell dose column:*** IT: intrathecal, ITu: intratumoral*; **Best clinical outcome column***: avg: average, CR: complete response, ctrls: controls, mths: months, PR: partial response, SD: stable disease, wks: weeks, yrs: years.

Supplementary Table 2: Cell Therapy with NK cells

Year(s)	Pt(#)	Diagnosis	Treatment	Cell dose	Best clinical outcomes
2004[23]	9	Recurrent malignant glioma	auto NK cells with IL-2 and IFN-β	$0.6\text{-}6.5x10^9$ (IV and ITu)	3 pts: PR<br>2 pts: MR
2020[102]	12	Recurrent MB and EPN	auto NK cells	$0.01\text{-}3x10^8/m^2$ (ICV)	1 pt: tumor regression for 3 wks<br>1pt: SD for 1 mth
2021[103]	14	Recurrent GBM	auto NK cells	$2\text{-}6x10^9$ (IV)	5 pts: tumor regressions for 28 – 76 mths
2023[104]	5	Recurrent GBM and grade 4 MB	haplo NK cells	$0.02\text{-}1x10^8$ (IT)	2 pts: SD for 40 days and 3 mths
2023[105]	9	Recurrent HER2+ GBM	irradiated HER2-CAR NK92 cell line	$0.1\text{-}1x10^8$ (ITu)	5 pts: SD for 7-37 wks
2025[106]	9	Recurrent/ refractory malignant glioma, DIPG, EPN, PB	allo NK cells	$4\text{-}5x10^7$ (IT)	1 pt: PR at 18mth follow-up<br>3 pts: SD at 18mth follow-up

Pt(#): Number of patients, *Diagnosis column:* DIPG: diffuse intrinsic pontine glioma, EPN: ependymoma, GBM: glioblastoma, HER2: human epidermal growth factor receptor 2, MB: medulloblastoma, PB: pineoblastoma; *Treatment column:* allo: allogeneic, auto: autologous, CAR: chimeric antigen receptor, haplo: haploidentical, IFN: interferon, IL: interleukin, NK: natural killer; *Cell dose column:* ICV: intraventricular, IT: intrathecal, ITu: intratumoral, IV: intravenous; *Best clinical outcome column*: MR: minor response, mths: months, PR: partial response, SD: stable disease, wks: weeks.
