# Supplementary material for: Cell therapy for brain tumors: The first 60 years
Source: Cell Rep Med. 2026 Feb 17;7(2):102626. doi: 10.1016/j.xcrm.2026.102626 (PMC12923960; doi:10.1016/j.xcrm.2026.102626)
Supplement: Document S2. Article plus supplemental information [file mmc2.pdf]

Cell Reports Medicine

CellPress
OPEN ACCESS

Review

Cell therapy for brain tumors: The first 60 years

Sanya Mehta,[1,2] Giedre Krenciute,[1,*] and Stephen Gottschalk[1,*]
[1]Department of Bone Marrow Transplantation and Cellular Therapy, St. Jude Children's Research Hospital, Memphis, TN 38105, USA
[2]Graduate School of Biomedical Sciences, St. Jude Children's Research Hospital, Memphis, TN 38105, USA
*Correspondence: giedre.krenciute@stjude.org (G.K.), stephen.gottschalk@stjude.org (S.G.)
https://doi.org/10.1016/j.xcrm.2026.102626

SUMMARY

Primary brain tumors remain among the most lethal cancers, but immunotherapy holds immense potential to overcome limitations of current standard treatment modalities. Since the late 1960s, early-phase clinical trials have iteratively tested cellular immunotherapies for the treatment of brain tumors. Six decades ago, in the earliest studies, brain tumor patients were treated with infusions of nonspecific leukocytes, peripheral blood mononuclear cells (PBMCs), and bone marrow cells. These earliest studies demonstrated safety and occasional durable antitumor responses, particularly when cell therapies were combined with conventional modalities or administered in the upfront setting. These early cell therapy approaches were chronologically followed by lymphokine-activated killer (LAK) cells, tumor-infiltrating lymphocytes (TILs), *ex vivo* nonspecifically expanded and antigen-specific T cells, natural killer (NK) cells, and chimeric antigen receptor (CAR) T cells. In this historical review, we summarize the clinical experience with adoptive cell therapies for brain tumors and review key findings from published clinical studies.

INTRODUCTION

Primary brain tumors remain among the leading causes of morbidity and mortality across all age groups.[1,2] Rickman J. Godlee performed the first documented successful resection of a primary brain tumor in 1884.[3] Since then, many advances have been made in neurosurgical procedures, radiation therapy, chemotherapy, and the molecular understanding of distinct brain tumor entities.[4] Yet, outcomes for many brain cancers, such as glioblastoma (GBM) and diffuse midline gliomas (DMGs), remain extremely poor.[5,6] Furthermore, acute and long-term toxicities of current treatment modalities, which compromise quality of life, remain a major limitation.[7]

To address these limitations, immunotherapy has emerged as a promising tool to specifically activate immune cells against cancer antigens. Adoptive cell therapy is a type of immunotherapy that is based on the infusion of disease-targeting allogeneic or autologous immune cells into patients.[8] The study of adoptive cell transfer dates back to 1913, when it was observed that inoculating rats with a mixture of sarcoma cells and splenocytes led to delayed tumor growth.[9] Since then, as a result of extensive laboratory and clinical testing, cell therapy has revolutionized the treatment for relapsed/refractory B cell malignancies.[10,11] Meaningful results have also been achieved for patients with late-stage melanoma and refractory synovial sarcoma.[12,13] For the past 6 decades, cellular immunotherapies have similarly been tested in the clinic for the treatment of primary brain tumors (Figure 1).[14–28] In this article, we review the approaches that have been tested and summarize the key takeaways from published clinical studies. While one could ask, why write a review on the history of cell therapy for brain tumors, we believe that it is as important to look back as it is to look forward. As the reader will appreciate, clinical response, albeit inconsistent, has been observed since the first cell therapy studies for brain tumors were conducted, and this insight alone puts the current results of chimeric antigen receptor (CAR) T cell therapy studies for brain tumors into perspective. As Mark Twain noted, "history doesn't repeat itself, but it often rhymes."

IMMUNOTHERAPY WITH LEUKOCYTES, PBMCs, AND BONE MARROW CELLS

Between 1968 and 2001, more than 200 brain tumor patients were treated with leukocytes, bone marrow cells as adjuvant immunotherapy, or peripheral blood mononuclear cells (PBMCs), and the results from these studies are summarized in Table 1 and reviewed in the following text. In the 1960s, there was convincing evidence that the immune system can recognize tumor cells via the existence of tumor neoantigens.[14] Lymphoid infiltration of brain tumors had been described and proposed as a predictor of favorable prognosis.[14] While the natural antitumor immune response is weak and easily overcome, mouse experiments have shown that immunization via "pre-transplantation" of brain tumors to any part of the body could break immune tolerance, leading to the rejection of otherwise lethal intracerebral glioblastoma grafts.[14] On the other hand, administration of immunosuppressive agents such as corticosteroids was known to impair immune function and even facilitate tumor growth.[14] Finally, today it is well recognized that systemically administered immune checkpoint inhibitors can have therapeutic effects on brain metastases and biological effects against GBMs.[29,30] However, at the time, it was thought that antibodies cannot cross the blood-brain barrier, so an immunotherapy for the treatment of brain tumors

© 2026 The Authors. Published by Elsevier Inc.
This is an open access article under the CC BY license (http://creativecommons.org/licenses/by/4.0/).

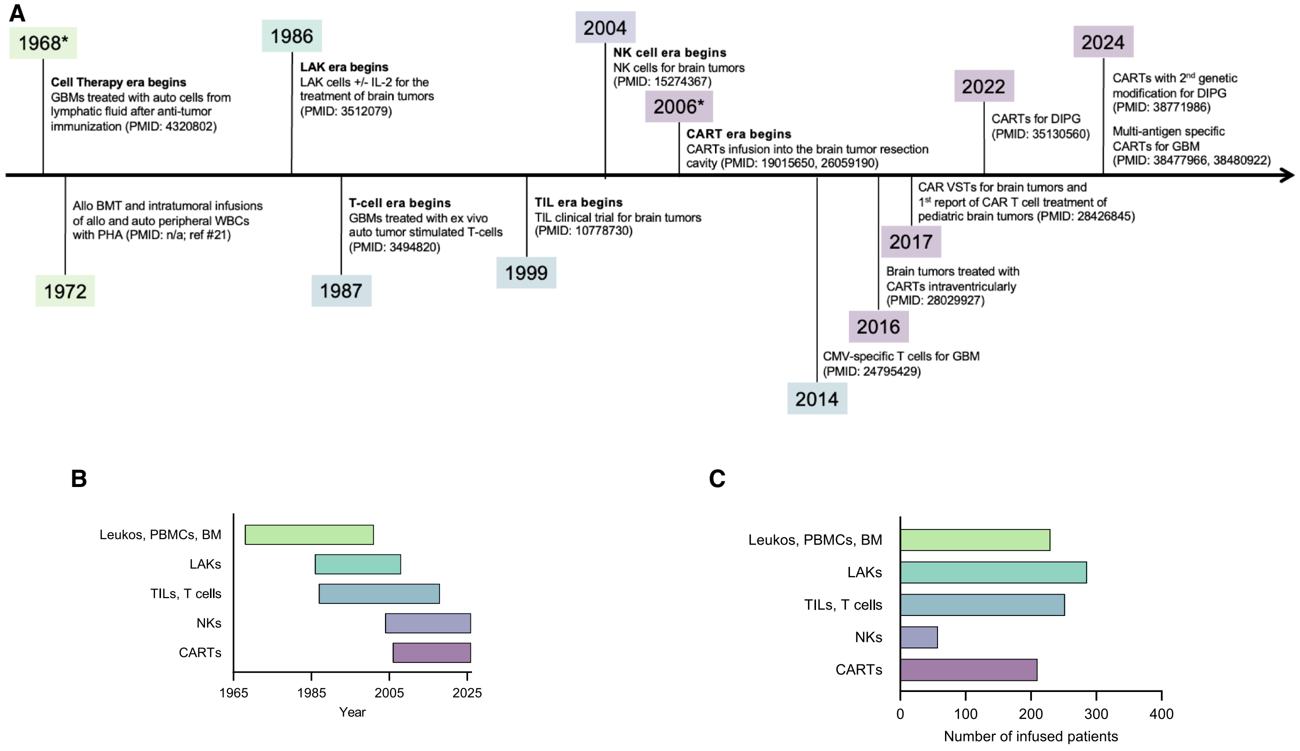

Figure 1. The 60-year evolution of cell therapy trials for brain tumors

(A) A timeline of key clinical milestones in cellular immunotherapy for brain tumors; year of publication is shown unless the year of infusion was reported in the publication (denoted by *). PubMed Identifier, PMID; for the 1972 study with no PMID, see Takakura et al.[21]

(B) A timeline representing the progression of clinical implementation of major cell therapy products for brain tumors (Leukos, PBMCs, BM, leukocytes, peripheral blood mononuclear cells, bone marrow; LAKs, lymphokine-activated killer cells; TILs, tumor-infiltrating lymphocytes; NKs, natural killer cells; CARTs, chimeric antigen receptor T cells).

(C) Total number of patients infused with the different major cell therapy products in clinical trials for brain tumors.

should be cell based.[14] This knowledge was translated into the first clinical cell therapy protocol for brain tumors in 1968.[14,31]

In this inaugural attempt to induce "allergic encephalotumoritis" in humans, three patients' primary tumors were resected, and bilateral tumor grafts were implanted subcutaneously into the mid-thighs. The tumor graft on the right thigh was boosted with the addition of Bacillus Calmette-Guérin (BCG) injections. Fourteen to 21 days after this procedure, immunity to the tumor grafts was confirmed by performing an intradermal reaction test using cryopreserved tumor homogenate. Once the patient was considered sensitized, lymphatic fluid was harvested via catheterization of the thoracic duct. Cells from the lymphatic fluid were concentrated, and 6–22 billion cells were injected near the primary tumor site, either intratumorally or into the subarachnoid space.[14,31]

Following this extensive protocol, one of the patients with a grade 2/3 oligoastrocytoma achieved a complete response (CR) with no relapse or progression during 17 months of follow-up. The other two patients with grade IV GBMs had progressive disease. However, in one of the patients, histological analysis of a reoperation specimen post therapy revealed the presence of a "thick lymphocyte crown" evident of a cellular immunological reaction surrounding the primary tumor. Altogether, the authors concluded that while the therapy was safe, the overall efficacy of this treatment regimen was limited.[14,31]

Ten other published clinical trials followed between 1972 and 2001 to evaluate the use of autologous and allogeneic leukocytes, bone marrow cell transfusions, and PBMCs.[21,32–45] The majority of the patients had relapsed or refractory GBMs or anaplastic astrocytomas, although in 5 of the studies, several patients were treated in the upfront setting prior to relapse.[21,32,34–36,42,43] The most promising clinical outcomes were produced by two studies.[33,41] In the first study, 17 recurrent grade III astrocytoma or GBM patients who had failed upfront radiotherapy and/or chemotherapy were treated with one or more infusions of autologous PBMCs into the tumor bed. One GBM patient, who was comatose at the time of infusion, had a sustained tumor regression and returned to full independence for 17 months post immunotherapy. Seven other patients had sustained neurological improvements and prolonged survival compared to historical controls.[33] In the second study, a total of 97 patients with recurrent malignant grade II–IV gliomas were treated with autologous lymphocytes. The lymphocytes were stimulated *ex vivo* with phytohemagglutinin (PHA) and interleukin (IL)-2 and then infused into the tumor bed following surgical debulking. Following immunotherapy, 76 patients had a positive initial response defined as either tumor regression or stable disease (SD) for at least 2 months after treatment. Eighteen of these responding patients, including 8 grade III

CellPress
OPEN ACCESS

Table 1. Cell therapy with leukocytes, PBMCs, and bone marrow cells

Year(s)	Pt (#)	Diagnosis	Treatment	Cell dose	Best clinical outcomes
1969,[14] 1970[31]	3	2 grade 4 GBM<br>1 grade 2/3 OA	auto cells from lymphatic fluid after antitumor immunization	6–22 × 10^9 (i.t. and SA)	OG pt: complete remission with no relapse for 17 mths<br>1 GBM pt: transient immune reaction at primary tumor site
1972,[21] 1975[32]	21	recurrent tumors<br>*pediatric:* MB, GBM, OPG, pinealoma, A, DIPG<br>*adult:* GBM, A, EPN, OG	adjuvant<br>*pediatric:* allo BM cells<br>*adult:* allo or auto leukos	1–9 × 10^9 (BM: i.v.; other: i.t.)	significantly higher survival compared to historical controls
1977[33]	17	recurrent grade 3–4 GBMs	auto PBMCs	0.01–1 × 10^9 (i.t.)	1 pt: sustained regression for 17 mths<br>7 pts: sustained neurological improvements and prolonged survival compared to historical controls
1978[34]	4	primary grade 3–4 GBMs after surgery and radiation	auto PBMCs	0.001–5 × 10^9 (IT)	1 pt: transient improvement in speech and reduction in tumor burden<br>1 pt: transient reduction in tumor burden
1979[35]	10	primary or recurrent GBM or grade 4 A after surgery, XRT, and/or chemotherapy	allo tumor RNA-sensitized leukos	0.7–1.5 × 10^9 (i.v.)	extended survival or delayed recurrence compared to conventional therapy
1981[36]	9	primary GBM, EPM, DIPG, OG, A (after surgical resection)	auto PBMCs	0.01–1 × 10^9 (i.t.)	significant improved survival compared to historical controls (avg: 23 vs. 10.3 mths)
1984[37]	4	recurrent GBM	auto PBMCs	0.08–4 × 10^9 (i.t.)	no benefit
1987[38]	18	recurrent GBM	auto PBMCs labeled with indium-111	2.1–6.7 × 10^9 (IT)	no benefit
1987,[39,40] 1990[41]	97	recurrent grades 2–4 glioma	auto PBMCs stimulated with PHA/IL-2	0.1–5 × 10^9 (i.t.)	18 pts: no recurrence for >6–33 mths incl grade 3 (n = 8) and 5 (n = 4) tumors<br>76 pts: tumor regression or stable disease for at least 2 mths
1989,[42] 1991[43]	31	GBMs following initial resection	auto PBMCs mixed with 3 × 10^6 IU IFN-α	mean 8.6 × 10^8 (i.t.)	1 pt: transient regression for 6 mths<br>2 pts: stable disease for 5–6 mths
1989[44]	4	MM, A, GBM	auto PBMCs stimulated with auto tumor cells	0.2–1.3 × 10^8 (i.v. or i.t.)	1 pt: neurological improvement
2001[45]	12	refractory GBM or A	auto PBMCs stimulated with OK-432	1–11.2 × 10^7 (i.t.)	2 pts: transient stable disease<br>2 pts: transient minor regressions

Pt (#), number of patients; A, astrocytoma; DIPG, diffuse intrinsic pontine glioma; EPN, ependymoma; GBM, glioblastoma; MB, medulloblastoma; MM, metastatic meningioma; OA, oligoastrocytoma; OG, oligodendroglioma; OPG, optic glioma; XRT, radiation therapy; allo, allogeneic; auto, autologous; BM, bone marrow; IFN, interferon; IL, interleukin; leuko, leukocyte; OK-432, lyophilized *Streptococcus pyogenes*; PBMCs, peripheral blood mononuclear cells; PHA, phytohemagglutinin; IT, intrathecal; i.t., intratumoral; i.v., intravenous; SA, subarachnoid; avg, average; incl, including; mths, months; pt, patient.

and 5 grade IV tumors, remained free of recurrence during follow-up (6–33 months).[41]

Promising results were also observed in clinical studies of adjuvant bone marrow cell or peripheral leukocyte infusions, which were from an allogeneic source for the majority of patients.[21,32] Treated patients had a significantly improved survival compared to historical controls, and one GBM patient remained alive without recurrence during the 5-year follow-up period.[32] However, many patients received their cell infusion in the upfront setting alongside conventional surgery, radiation, and chemotherapy regimens, making assessment of its benefit difficult. In addition, not all studies reported clinical benefits.[37,38] For example, the efficacy of an intrathecal injection of autologous leukocytes was compared with/without radiotherapy in 11 patients with recurrent GBM after resection. Five patients received only leukocytes and 6 leukocytes and radiation therapy, respectively. Patients who received only leukocytes had a shorter mean survival than patients who also received radiation therapy (4 vs. 11.4 months), highlighting that unspecific leukocytes do not have sufficient antitumor activity to prevent progression.[38]

In conclusion, these early clinical trials began to demonstrate that adoptive cell therapy for brain tumors is safe and beneficial in a subset of patients who failed frontline conventional therapies. However, with less than 10%, CRs in the non-adjuvant setting were uncommon.[14,41]

LYMPHOKINE-ACTIVATED KILLER CELL THERAPY

In 1981, it was first reported that PBMCs from cancer patients could be activated and rapidly expanded to large numbers *in vitro* by culturing them with IL-2 for a minimum of 2 days.[46] The resulting cell product was coined lymphokine-activated killer (LAK) cells and consisted of a mixture of natural killer (NK) cells, T cells, and NK T cells. LAK cells can lyse otherwise NK cell-resistant autologous tumor cells (albeit at very high effector to target ratios) but do not kill healthy cells.[47] Following encouraging preclinical and clinical findings in the setting of pulmonary and hepatic metastases,[48,49] the first use of LAK cells and/or IL-2 in the treatment of human brain tumors was reported in 1986,[15,50] and until 2008, close to 300 patients were treated with LAK cells, which represents the largest group of patients treated with cellular immunotherapy. The results from these studies are summarized in Table S1 and reviewed in the following text.

In the first study, patients with progressive malignant glioma were treated with IL-2 with or without autologous LAK cells intratumorally at the time of surgical resection. While there were no objective antitumor responses, this trial demonstrated the feasibility and safety of locoregional LAK cell infusions. Since this original report, at least 20 early-phase clinical studies were conducted to evaluate the use of LAK cells for brain tumors.[15,50–81] The majority of enrolled patients were GBMs and grade III astrocytomas in the relapsed/refractory setting. However, in at least 5 studies, LAK cells were administered in the upfront setting alongside or immediately following conventional surgical resection, chemotherapy, and/or radiation therapy.[53,57,65,66,74,77,78,80]

The best clinical responses after LAK cell therapy were observed in upfront studies where the patients were treated prior to relapse and in studies where relapsed/refractory patients were treated with LAK cells in the adjuvant setting alongside chemotherapy and/or other immunotherapies.[62,65,66,76] In one case report, a relapsed GBM patient's tumor continued to rapidly progress despite re-irradiation, interferon (IFN)-β, and chemotherapy with ANCU (1-(4-amino-2-methyl-5-pyrimidinyl) methyl-3-(2- chloroethyl)-3-nitrosourea hydrochloride). Intratumoral infusions of autologous LAK cells were then added to the treatment regimen, and after 9 courses of LAK cells, IFN-β, and ANCU, the patient achieved a CR, which was sustained (last follow-up: >9 years).[62,76] Promising results were also observed in a study in which 10 patients were treated intratumorally with autologous LAK cells alone, and 10 patients were treated with autologous LAK cells that were pre-incubated with a bispecific antibody prior to infusion. The bispecific antibody consisted of a CD3 monoclonal antibody (mAb) chemically conjugated to a glioma-targeting mAb. At the time of the study, the target antigen of the glioma-targeting mAb was unknown but was later identified to be neural cell adhesion molecule (NCAM, CD56).[82] All but one patient were treated after initial tumor resection and completion of radiation therapy and/or chemotherapy. One CR was observed in the group treated with LAK cells alone, and 4 CRs in the group that received bispecific antibody pre-incubated LAK cells, respectively.[65,66]

However, LAK cells did not produce a consistent clinical benefit in any of the conducted clinical studies.[15,50,67–69,72] While the reasons for inconsistent benefit remain largely unknown, one study highlighted that use of steroids during the month before leukapheresis was associated with a poor response to LAK cell therapy.[80] Likewise, high postsurgical tumor burden was associated with a poor response.[56] Finally, an autopsy study post LAK cell therapy demonstrated necrosis and infiltrating T cells close at the site of LAK cell administration post-surgical tumor resection but not at distant tumor sites within the brain, suggesting an inability of LAK cells to destroy macroscopic, non-resected tumor sites or their inability to migrate to tumor sites.[68]

In conclusion, while some studies reported favorable outcomes in a subset of patients, the overall efficacy of LAKs with IL-2 in brain tumor patients was limited. Thus, a randomized phase 2 study for brain tumor patients was never conducted, and LAK cells were eventually eclipsed by more targeted and potent cell therapy approaches.

TILs AND NONSPECIFIC AND ANTIGEN-SPECIFIC T CELLS

Between 1987 and 2018, more than 250 patients have been treated with *ex-vivo*-expanded tumor-infiltrating lymphocytes (TILs), non-specifically expanded T cells, or antigen-specific T cells, and the results from these studies are discussed in the following text and summarized in Table 2. In the quest to generate therapeutic cells with better antitumor activity than LAK cells, TILs were first identified in 1986.[83] TILs consist primarily of autologous cytotoxic T cells that are isolated from tumor specimens and expanded *ex vivo* in the presence of IL-2 prior to re-infusion. *In vivo*, TILs were 50–100 times more potent than LAK cells in pulmonary and hepatic metastatic tumor models.[83] An additional advantage of TILs is the presence of subpopulations of lymphocytes that can infiltrate growing

CellPress
OPEN ACCESS

Table 2. Cell therapy with tumor-infiltrating lymphocytes and nonspecific and antigen-specific T cells

Year(s)	Pt (#)	Diagnosis	Treatment	Cell dose	Best clinical outcomes
1987[22]	5	primary or recurrent AO or GBM	auto T cells after *in vitro* antitumor immunization	5×10^7 (i.t.)	2 pts: PR for 20 weeks and >24 mths
1996[85]	15	recurrent GBM or grade 3 A	auto T cells with IL-2 after antitumor + BCG immunization	0.1–9 $\times 10^{10}$ (i.v.)	10 pts: SD for 3 to >40 mths
1996,[84] 1999[16]	6	recurrent malignant glioma	auto TILs and IL-2	1×10^9 (i.t.)	1 pt: CR for >45 mths<br>2 pts: PR for >47 mths<br>2 pts: PR for 6 mths
1997[86]	5	recurrent grade 3–4 glioma	allo T cells after auto HLA sensitization and IL-2	0.1–5.15 $\times 10^9$ (i.t.)	2 pts: no reoccurrence for >28 mths<br>1 pt: SD for >28 mths
1998[87]	10	recurrent GBM or grade 3 A	auto T cells after antitumor + GM-CSF immunization	0.009–1.5 $\times 10^{11}$ (i.v.)	1 pt: no recurrence for >12 mths
1999[88]	4	recurrent GBM or anaplastic A	auto T cells after *in vitro* antitumor immunization	0.7–7.3 $\times 10^7$ (i.t.)	3 pts: tumor regression for 1 mth, 2 mths, and >1 year
2000[89]	12	grade 2 A, anaplastic glioma, GBM after surgery and XRT	auto T cells after antitumor + GM-CSF immunization	0.6–5.5 $\times 10^{10}$ (i.v.)	4 pts: PR for 11 to >29 mths<br>2 pts: SD for >20 and >27 mths
2000[90]	9	recurrent GBM or grade 3 A	auto T cells with IL-2 after antitumor + BCG immunization	1–9.6 $\times 10^{10}$ (i.v. or IA)	2 pts: CR for >4 and 5 years
2000[91]	19	recurrent GBM or grade 3 A	auto T cells after antitumor + GM-CSF immunization	0.5–5 $\times 10^{11}$ (i.v.)	1 pt: CR for >17 mths<br>7 pts: PR for 3 to >28 mths<br>2 pts: SD for >7 mths
2003[92]	10	recurrent GBM, anaplastic A, anaplastic OA	auto T cells after *in vitro* antitumor immunization	0.03–2.47 $\times 10^9$ (i.v.)	1 pt: CR for 21 mths<br>4 pts: PR for 1–16 mths
2008[93]	3	refractory anaplastic A or EPN	auto T cells after antitumor immunization	unknown (i.v.)	2 pts: tumor regression for 480 days and >7 years
2010[94]	5	primary GBM	adjuvant auto T cells	1–1.7 $\times 10^{10}$ (i.v.)	3 pts: CR for 5, 12, and >14 years<br>2 pts: PR for 24 and 39 mths
2012[95]	1	recurrent GBM	auto CMV-specific $CD8^+$ T cells followed with TMZ after 10+ days	4×10^7 (i.v.)	tumor regression for >17 mths
2014[19]	11	recurrent GBM	auto CMV-specific T cells	2.5–4 $\times 10^7$ (i.v.)	4 pts: SD for >5 mths to >4 years
2017[96]	23	primary or recurrent GBM, anaplastic OG, anaplastic OA, LGG, anaplastic EPN	auto lymphokine-activated T cells +/− TMZ	6.54–9.34 $\times 10^7$ (i.v.)	10 pts (+TMZ): 3 PR, 7SD<br>4 pts (−TMZ): 1 PR, 3 SD
2017[97]	91	primary GBM	auto T cells + TMZ	0.12–1.96 $\times 10^{10}$ (i.v.)	significant improved PFS compared to TMZ-only ctrl group (avg: 8.1 vs. 5.4 mths)
2018[98]	23	recurrent GBM	auto T cells after cancer/testis antigen-expressing T helper cell immunization	0.0054–1.86 $\times 10^9$ (i.v.)	3 pts: tumor regressions for >14, >22, and >27 mths

Pt (#), number of patients; A, astrocytoma; EPN, ependymoma; GBM, glioblastoma; LGG. low-grade glioma; OA, oligoastrocytoma; OG, oligodendroglioma; XRT, radiation therapy; allo, allogeneic; auto, autologous; BCG, Bacillus Calmette-Guerin; CMV, cytomegalovirus; GM-CSF, granulocyte-macrophage colony-stimulating factor; HLA, human leukocyte antigen; IL, interleukin; TILs, tumor-infiltrating lymphocytes; TMZ, temozolomide; IA, intraarterial; i.t., intratumoral; i.v., intravenous; avg, average; CR, complete response; ctrl, control; mths, months; PFS, progression-free survival; PR, partial response; SD, stable disease.

tumors, being thus potentially more capable of homing to metastatic tumor sites. While TILs have been evaluated in the clinic for other cancers,[12] leading to their Food and Drug Administration (FDA) approval for melanoma in 2024, only one clinical study for brain tumors has been published.[16,84] TILs were successfully expanded from the tumor specimens of all 6 patients and reinfused intratumorally. At long-term follow-up, one recurrent anaplastic astrocytoma patient had a CR (45 months), and two patients (recurrent astrocytoma or GBM) had sustained partial responses (PRs) (48 and 47 months).[16] Despite these encouraging results, no follow-up trials have been conducted in the US. Two phase 1 trials to evaluate TILs for brain tumors are

currently ongoing (NCT06640582 and NCT04943913), but the results have yet to be published.

In addition to TILs, other strategies with *ex-vivo*-expanded T cells have been evaluated for brain tumors in at least 16 early-phase clinical trials conducted between 1987 and 2018.[16,19,22,84–98] In these studies, about half of the patients have relapsed/refractory GBMs or grade III astrocytomas.[16,19,84–88,90–93,95,98] The other half had newly diagnosed GBMs or other malignant gliomas, and were treated concomitantly or following primary conventional therapies.[22,89,94,96,97] In two of the studies, autologous T cells were nonspecifically expanded from PBMCs using anti-CD3 and IL-2.[94,97] Both studies were randomized, treated newly diagnosed GBM patients, and gave multiple doses of autologous T cells intravenously. In one study, patients received T cells immediately following a standard regimen of maximum surgical resection, ANCU chemotherapy, and radiotherapy.[94] Three out of 5 patients had CRs lasting 5 to more than 14 years, and the median survival was 96.8 months longer than that of patients who only received the standard therapy regimen without T cells.[94] In the other study, patients received T cells simultaneously with standard temozolomide (TMZ) chemotherapy and radiotherapy following maximum surgical resection.[97] Median progression-free survival was significantly longer compared to patients who received TMZ and radiotherapy only (8.1 vs. 5.4 months), but there was no difference in overall survival.[97]

In the remaining studies, T cells were sensitized to tumor antigens using various strategies prior to or during *ex vivo* expansion.[16,19,22,84–93,95,96,98] In 6 of these studies, patients were immunized with an intradermal injection of their own irradiated tumor cells and an adjuvant, either granulocyte-macrophage colony-stimulating factor (GM-CSF) or BCG.[85,87,89–91,93] Following vaccination, T cells isolated from draining lymph nodes near the vaccine site or from PBMCs were activated and expanded *ex vivo* before intravenous infusion. In total, 56 relapsed/refractory and 12 newly diagnosed brain tumor patients were treated with this strategy. For relapsed/refractory patients, clinical activity was observed in 33 out 56 patients, including CRs (3), PRs/transient tumor regressions (15), and SD (15).[85,87,90,91,93] In the study with newly diagnosed patients, 6 out 12 patients had PRs (4) or SD (2).[89] While these findings are promising, especially for patients who have exhausted primary conventional treatments, a randomized phase 2 study has not been conducted.

Unlike the earlier clinical trials with leukocyte and LAK cell-based therapies, at least one patient in every T cell-based therapy trial experienced clinical improvement following immunotherapy.[16,19,22,84–98] Yet, like for LAK cell-based studies, potent and lasting antitumor responses remained rare. Reasons for the limited efficacy of cell-based immunotherapy started to emerge from preclinical studies.[99] In the 1970s, investigators had discovered that the endogenous T cell response requires the presentation of antigens on major histocompatibility complex (MHC) molecules.[100] Yet, in 2005, one study demonstrated by immunohistochemical staining of 88 primary astrocytic tumors that MHC class I expression was lost in 50% of the GBMs analyzed and that loss correlated with tumor grade.[101] Further, MHC class II expression was lost in 70% of GBMs, altogether identifying MHC downregulation as a major mechanism of immune evasion. The secretion of tumor-derived suppressive factors such as transforming growth factor (TGF)-β and prostaglandin E2 was also identified as a mechanism driving the reduced effectiveness of adoptively transferred cells.[99] T cell-based therapies have experienced a renaissance with the advent of genetic engineering, which is discussed in the CAR T cell therapy section of this review.

NK CELLS

Since 2004, about 50 patients have been treated with NK cell-based therapies, and the results from these studies are discussed in the following text and summarized in Table S2. Parallel to the rise of TILs and other T cell-based adoptive therapies, NK cell-based therapies emerged as a potential immunotherapeutic strategy for brain tumors. In the first NK cell clinical trial conducted for brain tumors, 9 patients received intravenous and intratumoral autologous NK cells together with IL-2 and IFN-β.[23] Three transient PRs (50% decrease) and 2 minor responses (25% decrease) were observed. In 3 of the responding patients, only the first NK cell dose induced tumor regression, suggesting outgrowth of an NK cell-resistant tumor cell population.[23] Five other NK cell therapy studies for brain tumors have been published.[23,102–106] One of these studies was the first to explore the use of genetically modified NK cells for brain tumor patients.[102,103,106] In this study, 9 patients with recurrent human epidermal growth factor receptor 2 (HER2)+ GBM were treated with repeated intratumoral infusions of irradiated off-the-shelf HER2-CAR NK-92 cells. Following treatment, 5 patients experienced temporary SD, which lasted between 7 and 37 weeks.[105] In total, transient tumor regressions were observed in 4 out of the 6 brain tumor-specific NK cell studies, but no CRs were observed.[102,103,106] NK cell therapy for brain tumors remains under active preclinical and clinical investigation, particularly since it has become feasible to readily genetically modify NK cells to enhance their antitumor activity.[107]

CAR T CELLS

While clinical responses were observed in the aforementioned early-phase cell therapy trials with antigen-specific T cells for brain tumors, the endogenous immune response was not sufficient to mount potent and lasting antitumor responses.[16,19,22,84–98] The advent of T cell engineering offered a potential solution to this roadblock by not only reliably generating brain tumor-specific T cells but also generating T cells with enhanced effector function. Initial genetic engineering approaches focused on expressing CARs in T cells to generate brain tumor-specific T cells. The original CAR structure was published in 1993 and consists of an antigen recognition domain, a hinge/transmembrane domain, and a cytoplasmic signaling domain.[8,108,109] The antigen-binding domain consists of a single-chain variable fragment (scFv) derived from a mAb or a ligand specific for a tumor-associated antigen expressed on the surface of tumor cells. This enables CAR T cells to recognize tumor cells in a non-MHC-restricted manner, rendering them resistant to immune evasion tactics of tumor cells, including downregulation of MHC.[8] The drawback of this design is

CellPress
OPEN ACCESS

that only the cell surface antigen can be targeted. However, the development of so-called "peptide-centric" CARs enables targeting of peptide derived from intracellular molecules presented by MHC molecules.[110] The cytoplasmic domain of CARs consists of signaling domains for T cell activation and costimulation, and the most common domains used are derived from CD3ζ for T cell activation and CD28 and/or 41BB (CD137) for costimulation.

CAR T cells targeting CD19 or BCMA have revolutionized the care of patients with relapsed/refractory B cell malignancies or multiple myeloma over the last 15 years, and currently, there are 7 FDA-approved CAR T cell products.[10,11,111] In contrast, early-phase clinical studies evaluating CAR T cells for solid tumors and brain tumors have shown limited activity unless in the setting of low disease burden.[112–114] This limited efficacy is most likely multifactorial and includes (1) low level and heterogeneous antigen expression, (2) limited expansion and persistence of CAR T cells, (3) limited homing to and penetration of tumor sites, and (4) the immunosuppressive tumor microenvironment. These roadblocks have been reviewed in several recent excellent reviews.[115–126] Since 2006, more than 200 patients with brain tumors have been infused with CAR T cells in 17 clinical studies, and we will focus here on reviewing clinical outcomes, which are summarized in Table 3.[17,18,20,24–28,112,127–142]

Monospecific CAR T cells targeting IL13Rα2, HER2, GD2, EGFR variants (vIII; E806), and EphA2 have been explored in early-phase clinical studies in addition to bispecific CAR T cells expressing (1) two CARs specific for IL13Rα2 and EGFR or (2) one CAR specific for EGFRvIII and one bispecific T cell engager specific for EGFR. Second genetic modifications to enhance safety (inducible caspase-9; iC9) or improve antitumor activity (constitutive active IL-7 receptor, C7R) of CAR T cells have also been explored.[25,28,127,138]

The first brain tumor patient was treated with CAR T cells targeting IL13Rα2 in 2006.[24] In this trial, 3 recurrent/refractory grade III or IV glioma patients were treated with repeated intratumoral injections of $CD8^+$ CAR T cell clones that expressed an IL13Rα2-CAR with a CD3ζ signaling domain.[17,24,128] While no objective antitumor responses were observed, this study illustrated that repeat CAR T cell administration to brain tumor patients was both feasible and safe. $CD8^+$ IL13Rα2-CAR T cell clones were explored in one follow-up study in which the glucocorticoid receptor was knocked out and clones were infused with IL-2 to boost their antitumor activity. However, only transient responses were observed in 2 out of 6 patients.[129]

The first major clinical successes of CAR T cells for brain tumors were observed in a large follow-up phase 1 trial.[20,140] In this trial, polyclonal T cells were evaluated that expressed an IL13Rα2-CAR with a 41BB.CD3ζ signaling domain to not only provide T cell activation but also provide costimulation. A total of 92 patients with recurrent high-grade glioma received IL13Rα2-CAR T cells either into the tumor cavity post resection and/or intracerebroventricularly in this trial between 2015 and 2020. Two patients achieved a CR (7.5 months; 3 years, ongoing), 2 patients achieved transient PRs, 10 patients achieved sustained SD lasting 3–15 months, 16 patients achieved transient SD lasting 51–90 days, and 62 patients had no response.[20,140] This trial is also the first published report of delivering cell therapy to brain tumors using intracerebroventricular (i.c.v.) injection. The trial initially opened as a two-arm study evaluating only intratumoral delivery following biopsy or resection. However, three additional arms involving i.c.v. or dual intratumoral/i.c.v. delivery were later added. The rationale to add i.c.v. dosing stemmed from early clinical experience on the trial involving a patient with recurrent, highly aggressive multifocal leptomeningeal GBM.[20] This patient initially received six weekly CAR T cell infusions into the resected cavity of the largest tumor, resulting in stabilization of the local treated site. However, during this initial treatment, the patient's nonresected tumor continued to progress, and multiple new brain and spine lesions appeared. The patient then received 10 additional i.c.v. CAR T cell injections, resulting in what appeared to be a complete elimination of all the patient's tumors, which persisted for 7.5 months. This clinical experience, combined with supporting preclinical data suggesting that i.c.v. delivery is more effective for trafficking cells to sites of multifocal disease, provides a strong rationale for further exploration of i.c.v. administration of CAR T cells.[20,143]

To date, findings from 18 ongoing and completed early-phase CAR T cell clinical trials for brain tumors have been published.[17,18,20,24–28,112,127–140,142] Clinical responses have been consistently observed in clinical studies targeting HER2, GD2, and B7-H3 in subset of patients, particularly for pediatric DMG after i.c.v. or intravenous infusion of autologous GD2-CAR or B7-H3-CAR T cells.[25,112,127,130] This led to a 2-fold increase in median survival in comparison to historical controls (∼20 vs. 10 months). In contrast, clinical responses were only observed in 1 out of 3 clinical studies targeting EGFRvIII.[133–136] While these monospecific CAR T cell therapy studies overall demonstrated safety, toxicities were also observed due to local inflammatory responses at the tumor sites that included pseudoprogression. These side effects were coined tumor inflammation-associated neurotoxicity (TIAN) to contrast them from the immune effector cell-associated neurotoxicity syndrome (ICANS) observed in patients after CAR T cell therapy, who do not have brain tumors.[144]

Three clinical studies have been conducted that explored a second genetic modification to improve CAR T cell function.[26–28,142] In two of the studies, different bispecific CAR T cells were infused: one expressing a 2nd CAR (EGFR and IL13Rα2) and the other expressing a CAR (EGFRvIII) and a bispecific T cell engager that recognizes EGFR and CD3ε.[26,27,142] In the third study, GD2-CAR T cells were genetically modified to express a constitutive active IL-7 cytokine receptor (C7R).[28] The safety and efficacy of i.c.v.-delivered EGFR/IL13Rα2-CAR T cells were evaluated in 18 adult patients with GBM.[27,142] Early-onset neurotoxicity was consistently observed, and several patients had clinical benefit, including one PR, one sustained SD (>7.7 months), and 9 transient SD (1–3 months).[27,142] Three patients with GBM have been reported after the i.c.v. administration of T cells expressing EGFRvIII-CARs and EGFR T cell engagers.[26] Early-onset neurotoxicity was observed in 2 out of 3 patients, and one patient had a sustained PR (>5 months) and two transient PRs, respectively.[26] Finally, the safety and efficacy of intravenously administered GD2-CAR T cells and GD2-CAR T cells expressing C7R (GD2-CAR.C7R) T cells were compared in one study for pediatric patients with DMG.[28] Three patients received GD2-CAR T cells, and eight received GD2-CAR.C7R T cells. Patients receiving GD2-CAR.C7R T cells had improvement from baseline neurologic

Table 3. Cell therapy with CAR T cells

Year(s)	Pt (#)	Diagnosis	Target	Treatment	Cell dose	Best clinical outcomes
**CD8$^+$ CAR T cell clones**						
2008,[17] 2015,[24] 2017[128]	3	recurrent GBM	IL13Rα2	Costim: no; LD: no other: HyTK transgene	0.1–1 × 10^8 (i.t.)	evidence of transient inflammation and targeting of IL13Rα2+ tu cells
2017,[128] 2022[129]	6	recurrent GBM		Costim: no; LD: no other: HyTK transgene; KO GR; IL2	0.2– 2 × 10^8 (i.t.)	2 pts: transient tumor necrosis
**Polyclonal CAR T cells**						
2023,[130] 2025[112]	21	DMG	B7-H3	Costim: 41BB T cells: CD4:CD8 ratio, 1:1 LD: no	0.1–1 × 10^7 (i.c.v.)	1 pt: transient PR (60 days) 15 pts: SD (2 to >37.5 mths)
2021[131]	1	recurrent GBM		Costim: 41BB T cells: CD25/ CD45RA depleted; LD: no	0.4–2 × 10^7 (i.t.)	tu reduction (50 days)
2025[132]	4	recurrent or refractory DMG, DHG, ATRT	EGFR806	Costim: 41BB T cells: CD4:CD8 ratio, 1:1 LD: no	1–2.5 × 10^7 (i.t. or i.c.v.)	1 DMG pt: SD followed by CR (2 years) to subsequent chemotherapy
2017,[133] 2021[134]	10	recurrent or progressive GBM	EGFRvIII	Costim: 41BB T cells: bulk; LD: no	1.75–5 × 10^8 (i.v.)	1 pt: sustained SD (15 mths) 4 pts: transient SD (1–2 mths)
2019[135]	18	recurrent GBM		Costim: CD28/41BB T cells: bulk; LD: yes other: IL2	1 × 10^7– 6 × 10^{10} (i.v.)	none
2024[136]	7	newly diagnosed GBM		Costim: 41BB T cells: bulk; LD: no other: anti-PD1	0.47–2 × 10^8 (i.v.)	none
2021[137]	3	recurrent GBM	EphA2	Costim: 41BB T cells: bulk; LD: yes	1 × 10^6/kg (i.v.)	1 pt: SD 3 mths
2022,[25] 2024[127]	12	H3K27M-mutant DMG, spinal DMG	GD2	Costim: 41BB T cells: bulk; LD: yes (pre i.v.) other: IC9 transgene	1–3 × 10^6/kg (i.v.) 1–3 × 10^7 (i.c.v.)	1 pt: CR (>30 mths) 1 pt: >30 mths (no change in tu vol) 3 pts: transient PR (>50%) 3 pts: transient tu vol reduction
2023[138]	8	recurrent or progressive GBM		Costim: CD28/41BB T cells: bulk; LD: yes other: iC9 transgene	2.5 × 10^6/kg (i.v.) 1 × 10^5/kg (i.t.)	4 pts: PR (2–24 mths) 1 pt: SD (4 mths)
2017[18]	17	progressive recurrent GBM	HER2	Costim: CD28 T cells: virus specific; LD: no	0.01–1 × 10^8/m^2 (i.v.)	1 pt: PR (9 mths) 7 pts: SD (8 weeks to 29 mths)
2021[139]	3	refractory anaplastic A, metastatic EPN		Costim: 41BB T cells: CD4:CD8 ratio, 1:1 LD: no	1–2.5 × 10^7 (i.t., i.c.v.)	1 pt: SD (<90 days)

(*Continued on next page*)

Table 3. *Continued*

Year(s)	Pt (#)	Diagnosis	Target	Treatment	Cell dose	Best clinical outcomes
2016,[20] 2024[140]	65	recurrent HGG	IL13Rα2	Costim: 41BB<br>T cells: TCM, TSCM<br>LD: no	0.02–2 × 10^8 (i.t., i.c.v.; i.t./i.c.v.)	2 pts: CR (7.5 mths, >43 mths)<br>2 pts: PR (1 mths, 9 mths)<br>10 pts: SD (3–15 mths)<br>16 pts: SD (51–90 days)
2025[141]	4	recurrent GBM	CLTX	Costim: CD28<br>T cells: TCM, TSCM<br>LD: no	0.4–2 × 10^7 (i.t.)	3 pts: SD (1–2 mths)
Polyclonal CAR T cells with genetic modification to enhance antitumor activity						
2024[26]	3	recurrent GBM	EGFRvIII EGFR*	Costim: 41BB<br>T cells: bulk; LD: no<br>other: T cell engager	1 × 10^7 (i.c.v.)	1 pt: PR lasting >5 mths<br>1 pt: transient PRs
2024,[27] 2025[142]	18	recurrent or progressive GBM	EGFR IL13Rα2	Costim: 41BB<br>T cells: bulk; LD: no<br>other: 2nd CAR	0.5–2.5 × 10^7 (i.c.v.)	1 pt: PR (3 mths),<br>2 pts: SD (>7.7 mths, >16.6 mths)<br>9 pts: SD (1–3 mths)
2024[28]	11	H3K27M-mutant DMG, MB, ATRT	GD2	Costim: 41BB<br>T cells: bulk; LD: yes<br>other: C7R transgene (8)	(i.v.)	2 pts: PR (>1 year, <20 weeks)<br>5 pts: SD (<30 weeks)<br>3 pts: transient neurological improvement for <3 weeks

Pt (#), number of patients; A, astrocytoma; ATRT, atypical teratoid/rhabdoid tumor; DHG, diffuse hemispheric glioma; DMG, diffuse midline glioma; EPN, ependymoma; GBM, glioblastoma; HGG, high-grade glioma; MB, medulloblastoma; *, *targeted with bispecific T cell engager*; B7-H3, CD276; EGFR806, epidermal growth factor receptor epitope defined by mAb 806 (cancer specific); EGFRvIII, EGFR variant III (cancer specific); EphA2, EPH receptor A2; GD2, disialoganglioside; HER2, human epidermal growth factor receptor 2; IL13Rα2, IL-13 receptor subunit alpha 2; CLTX, chlorotoxin; Costim, costimulatory domain of CAR; GR, glucocorticoid receptor; HyTK, hygromycin phosphotransferase gene/herpes simplex virus type 1 thymidine kinase fusion gene; iC9, inducible caspase-9 gene; KO, knockout; pre i.v., before i.v. infusion; i.c.v., intracerebroventricular; i.t., intratumoral; i.v., intravenous; CR, complete response; mths, months; PR, partial response; SD, stable disease; tu, tumor; vol, volume.

deficits (2 to >12 months), and PRs were observed in two patients. In contrast, in the GD2-CAR T cell patient cohort, benefits were limited.[28]

Correlative analyses from clinical trials of CAR T cell therapy for brain tumors have highlighted mechanisms of immune evasion, including the development of antigen-loss variants, adaptive changes in the tumor microenvironment such as an influx of myeloid cells and inhibitory regulatory T cells, and increased expression of inhibitory molecules such as PD-L1, PD-L2, PD1, TIM3, and indoleamine 2,3-dioxygenase 1 (IDO1).[20,25,130,133,134,136,138]

DISCUSSION AND OUTLOOK

Cellular therapy has been explored for brain tumors for the last six decades. Early clinical studies aimed to augment antitumor immune response with infusions of nonspecific leukocytes, PBMCs, bone marrow cells, LAK cells, and *ex-vivo*-activated NK- and T cells. Durable responses were rare, but CRs in a subset of patients were observed, especially when cell therapy was combined with conventional therapy or administered in the upfront setting. However, it is important to acknowledge that many of these studies were conducted using standardized response criteria, such as the McDonald Criteria,[145] which were developed prior to the widespread recognition of pseudoprogression.[146] Pseudoprogression refers to transient treatment-related effects such as enhanced tumor lesions, increased contrast enhancement, and edema that occur in around 20%–30% of brain tumor patients following chemoradiotherapy and/or immunotherapy.[146] These effects can mimic tumor growth on MRI, but unlike true tumor progression, these effects eventually subside without any change in therapy.[146] The recognition of pseudoprogression and advancements in imaging technologies led to the widespread implementation of the more rigorous Response Assessment in Neuro-Oncology Criteria for High-Grade Gliomas (RANO-HGG) recommendations, published in 2010,[147] and the updated RANO 2.0 criteria, published in 2023.[148] Enrollment of patients and interpretations of outcomes from earlier clinical studies were likely impacted by the limited recognition of pseudoprogression at the time. Thus, these earlier historical clinical outcomes should be contextualized in relation to the imaging technologies and response guidelines available

at the time, and caution should be exercised when directly comparing the findings to more recent clinical trials.

The advent of genetic engineering enabled the specific targeting of antigens expressed on brain tumor cells with CAR T cells. However, despite being able to directly target brain tumor cells, the clinical activity of CAR T cells has been limited. This lack of efficacy is most likely multifactorial and includes, but is not limited to, heterogeneous expression of the targeted antigens, limited ability of immune cells to traffic to and penetrate brain tumors, and the hostile tumor microenvironment. These roadblocks have been recently discussed in detail in several excellent review articles.[115–126] Despite the caveats of pseudoprogression, many lessons from the earlier cell therapy trials can be applied toward overcoming the barriers to effective CAR T cell therapies. For example, the best clinical responses from the early clinical studies were observed when patients were treated in the upfront setting prior to relapse or when relapsed/refractory patients were treated with cell therapy in the adjuvant setting alongside chemotherapy and/or other immunotherapies.[62,65,66,76,94,97] Currently, CAR T cells are being largely evaluated in the clinic as monotherapy for the treatment of relapsed/refractory CNS disease.[17,18,20,24–28,112,127–142] Thus, moving CAR T cells into the upfront treatment setting and incorporating combinatorial approaches hold promise to improve their efficacy. In addition, steroid use during the month before leukapheresis was associated with poor responses to LAKs.[80] High doses of corticosteroids are similarly known to negatively impact CAR T cells in preclinical models,[143,149] yet they are currently widely used to manage CNS edema before and during CAR T cell treatment.[17,18,20,24–28,112,127–142] Thus, investigation into alternative forms of edema management is warranted to preserve CAR T cell efficacy while managing baseline and treatment-related edema.

Altogether, counteracting these roadblocks with additional genetic modification of CAR T cells and combinatorial therapies holds the promise to improve their efficacy. Likewise, other immune cell subsets, including genetically engineered NK cells, warrant further exploration. We are hopeful that over the next two decades, these refined cell therapy approaches will induce durable remissions in pediatric and adult patients diagnosed with brain tumors.

ACKNOWLEDGMENTS

This work was supported by NINDS grant nos. R01NS121249 and R01NS122859 to G.K., NCI grant no. U01CA281823 to G.K., Alliance for Cancer Gene Therapy, Alex's Lemonade Stand Foundation (ALSF), and the American Lebanese Syrian Associated Charities to G.K. and S.G. The content is solely the responsibility of the authors and does not necessarily represent the official views of the National Institutes of Health.

AUTHOR CONTRIBUTIONS

S.M. wrote the first draft of the manuscript and designed the figure. S.M., G.K., and S.G. reviewed and edited the manuscript.

DECLARATION OF INTERESTS

G.K. and S.G. have patents or patent applications in the fields of cell or gene therapy for cancer. S.G. is a member of the Scientific Advisory Board of Be Biopharma and the Data and Safety Monitoring Board (DSMB) of Immatics.

SUPPLEMENTAL INFORMATION

Supplemental information can be found online at https://doi.org/10.1016/j.xcrm.2026.102626.

REFERENCES

1. Rouse, C., Gittleman, H., Ostrom, Q.T., Kruchko, C., and Barnholtz-Sloan, J.S. (2016). Years of potential life lost for brain and CNS tumors relative to other cancers in adults in the United States, 2010. Neuro Oncol. *18*, 70–77. https://doi.org/10.1093/neuonc/nov249.
2. Miller, K.D., Ostrom, Q.T., Kruchko, C., Patil, N., Tihan, T., Cioffi, G., Fuchs, H.E., Waite, K.A., Jemal, A., Siegel, R.L., and Barnholtz-Sloan, J.S. (2021). Brain and other central nervous system tumor statistics, 2021. CA Cancer J. Clin. *71*, 381–406. https://doi.org/10.3322/caac.21693.
3. Kirkpatrick, D.B. (1984). The first primary brain-tumor operation. J. Neurosurg. *61*, 809–813. https://doi.org/10.3171/jns.1984.61.5.0809.
4. Pollack, I.F., Agnihotri, S., and Broniscer, A. (2019). Childhood brain tumors: current management, biological insights, and future directions. J. Neurosurg. Pediatr. *23*, 261–273. https://doi.org/10.3171/2018.10.Peds18377.
5. Stupp, R., Mason, W.P., van den Bent, M.J., Weller, M., Fisher, B., Taphoorn, M.J.B., Belanger, K., Brandes, A.A., Marosi, C., Bogdahn, U., et al. (2005). Radiotherapy plus concomitant and adjuvant temozolomide for glioblastoma. N. Engl. J. Med. *352*, 987–996. https://doi.org/10.1056/NEJMoa043330.
6. Hoffman, L.M., Veldhuijzen van Zanten, S.E.M., Colditz, N., Baugh, J., Chaney, B., Hoffmann, M., Lane, A., Fuller, C., Miles, L., Hawkins, C., et al. (2018). Clinical, Radiologic, Pathologic, and Molecular Characteristics of Long-Term Survivors of Diffuse Intrinsic Pontine Glioma (DIPG): A Collaborative Report From the International and European Society for Pediatric Oncology DIPG Registries. J. Clin. Oncol. *36*, 1963–1972. https://doi.org/10.1200/jco.2017.75.9308.
7. Angoumis, K., Padilla, C.S., Kouwenhoven, M.C.M., Bijlsma, R.M., Kaal, S.E.J., Tromp, J.M., Bos, M.E.M.M., van der Hulle, T., Broen, M.P.G., Nuver, J., et al. (2025). Adverse health outcomes and health-related quality of life (HRQoL) among long-term adolescent and young adult (AYA) brain tumour survivors: results from the population-based SURVAYA study. Support. Care Cancer *33*, 95. https://doi.org/10.1007/s00520-025-09155-9.
8. Singh, A.K., and McGuirk, J.P. (2020). CAR T cells: continuation in a revolution of immunotherapy. Lancet Oncol. *21*, e168–e178. https://doi.org/10.1016/s1470-2045(19)30823-x.
9. Woglom, W.H. (1933). Absorption of the Protective Agent from Rats Resistant to a Transplantable Sarcoma. Am. J. Cancer *17*, 873–893. https://doi.org/10.1158/ajc.1933.873.
10. Gardner, R.A., Finney, O., Annesley, C., Brakke, H., Summers, C., Leger, K., Bleakley, M., Brown, C., Mgebroff, S., Kelly-Spratt, K.S., et al. (2017). Intent-to-treat leukemia remission by CD19 CAR T cells of defined formulation and dose in children and young adults. Blood *129*, 3322–3331. https://doi.org/10.1182/blood-2017-02-769208.
11. Maude, S.L., Frey, N., Shaw, P.A., Aplenc, R., Barrett, D.M., Bunin, N.J., Chew, A., Gonzalez, V.E., Zheng, Z., Lacey, S.F., et al. (2014). Chimeric antigen receptor T cells for sustained remissions in leukemia. N. Engl. J. Med. *371*, 1507–1517. https://doi.org/10.1056/NEJMoa1407222.
12. Sarnaik, A.A., Hamid, O., Khushalani, N.I., Lewis, K.D., Medina, T., Kluger, H.M., Thomas, S.S., Domingo-Musibay, E., Pavlick, A.C., Whitman, E.D., et al. (2021). Lifileucel, a Tumor-Infiltrating Lymphocyte Therapy, in Metastatic Melanoma. J. Clin. Oncol. *39*, 2656–2666. https://doi.org/10.1200/jco.21.00612.

13. D'Angelo, S.P., Araujo, D.M., Abdul Razak, A.R., Agulnik, M., Attia, S., Blay, J.Y., Carrasco Garcia, I., Charlson, J.A., Choy, E., Demetri, G.D., et al. (2024). Afamitresgene autoleucel for advanced synovial sarcoma and myxoid round cell liposarcoma (SPEARHEAD-1): an international, open-label, phase 2 trial. Lancet *403*, 1460–1471. https://doi.org/10.1016/s0140-6736(24)00319-2.

14. Trouillas, P., and Lapras, C. (1969). Cellular immunotherapy of cerebral glioblastoma. Apropos of 2 results]. J. Med. Lyon *50*, 1269–1291.

15. Jacobs, S.K., Wilson, D.J., Kornblith, P.L., and Grimm, E.A. (1986). Interleukin-2 or autologous lymphokine-activated killer cell treatment of malignant glioma: phase I trial. Cancer Res. *46*, 2101–2104.

16. Quattrocchi, K.B., Miller, C.H., Cush, S., Bernard, S.A., Dull, S.T., Smith, M., Gudeman, S., and Varia, M.A. (1999). Pilot study of local autologous tumor infiltrating lymphocytes for the treatment of recurrent malignant gliomas. J. Neuro Oncol. *45*, 141–157. https://doi.org/10.1023/a:1006293606710.

17. Yaghoubi, S.S., Jensen, M.C., Satyamurthy, N., Budhiraja, S., Paik, D., Czernin, J., and Gambhir, S.S. (2009). Noninvasive detection of therapeutic cytolytic T cells with 18F-FHBG PET in a patient with glioma. Nat. Clin. Pract. Oncol. *6*, 53–58. https://doi.org/10.1038/ncponc1278.

18. Ahmed, N., Brawley, V., Hegde, M., Bielamowicz, K., Kalra, M., Landi, D., Robertson, C., Gray, T.L., Diouf, O., Wakefield, A., et al. (2017). HER2-Specific Chimeric Antigen Receptor-Modified Virus-Specific T Cells for Progressive Glioblastoma: A Phase 1 Dose-Escalation Trial. JAMA Oncol. *3*, 1094–1101. https://doi.org/10.1001/jamaoncol.2017.0184.

19. Schuessler, A., Smith, C., Beagley, L., Boyle, G.M., Rehan, S., Matthews, K., Jones, L., Crough, T., Dasari, V., Klein, K., et al. (2014). Autologous T-cell therapy for cytomegalovirus as a consolidative treatment for recurrent glioblastoma. Cancer Res. *74*, 3466–3476. https://doi.org/10.1158/0008-5472.Can-14-0296.

20. Brown, C.E., Alizadeh, D., Starr, R., Weng, L., Wagner, J.R., Naranjo, A., Ostberg, J.R., Blanchard, M.S., Kilpatrick, J., Simpson, J., et al. (2016). Regression of Glioblastoma after Chimeric Antigen Receptor T-Cell Therapy. N. Engl. J. Med. *375*, 2561–2569. https://doi.org/10.1056/NEJMoa1610497.

21. TAKAKURA, K., MIKI, Y., KUBO, O., OGAWA, N., MATSUTANI, M., and SANO, K. (1972). Adjuvant Immunotherapy for Malignant Brain Tumors. Jpn. J. Clin. Oncol. *2*, 109–120. https://doi.org/10.1093/oxfordjournals.jjco.a039811.

22. Kitahara, T., Watanabe, O., Yamaura, A., Makino, H., Watanabe, T., Suzuki, G., and Okumura, K. (1987). Establishment of interleukin 2 dependent cytotoxic T lymphocyte cell line specific for autologous brain tumor and its intracranial administration for therapy of the tumor. J. Neuro Oncol. *4*, 329–336. https://doi.org/10.1007/bf00195603.

23. Ishikawa, E., Tsuboi, K., Saijo, K., Harada, H., Takano, S., Nose, T., and Ohno, T. (2004). Autologous natural killer cell therapy for human recurrent malignant glioma. Anticancer Res. *24*, 1861–1871.

24. Brown, C.E., Badie, B., Barish, M.E., Weng, L., Ostberg, J.R., Chang, W.C., Naranjo, A., Starr, R., Wagner, J., Wright, C., et al. (2015). Bioactivity and Safety of IL13Rα2-Redirected Chimeric Antigen Receptor CD8+ T Cells in Patients with Recurrent Glioblastoma. Clin. Cancer Res. *21*, 4062–4072. https://doi.org/10.1158/1078-0432.Ccr-15-0428.

25. Majzner, R.G., Ramakrishna, S., Yeom, K.W., Patel, S., Chinnasamy, H., Schultz, L.M., Richards, R.M., Jiang, L., Barsan, V., Mancusi, R., et al. (2022). GD2-CAR T cell therapy for H3K27M-mutated diffuse midline gliomas. Nature *603*, 934–941. https://doi.org/10.1038/s41586-022-04489-4.

26. Choi, B.D., Gerstner, E.R., Frigault, M.J., Leick, M.B., Mount, C.W., Balaj, L., Nikiforow, S., Carter, B.S., Curry, W.T., Gallagher, K., and Maus, M.V. (2024). Intraventricular CARv3-TEAM-E T Cells in Recurrent Glioblastoma. N. Engl. J. Med. *390*, 1290–1298. https://doi.org/10.1056/NEJMoa2314390.

27. Bagley, S.J., Logun, M., Fraietta, J.A., Wang, X., Desai, A.S., Bagley, L.J., Nabavizadeh, A., Jarocha, D., Martins, R., Maloney, E., et al. (2024). Intrathecal bivalent CAR T cells targeting EGFR and IL13Rα2 in recurrent glioblastoma: phase 1 trial interim results. Nat. Med. *30*, 1320–1329. https://doi.org/10.1038/s41591-024-02893-z.

28. Lin, F.Y., Stuckert, A., Tat, C., White, M., Ruggieri, L., Zhang, H., Mehta, B., Lapteva, N., Mei, Z., Major, A., et al. (2024). Phase I Trial of GD2.CART Cells Augmented With Constitutive Interleukin-7 Receptor for Treatment of High-Grade Pediatric CNS Tumors. J. Clin. Oncol. *42*, 2769–2779. https://doi.org/10.1200/jco.23.02019.

29. McFaline-Figueroa, J.R., Sun, L., Youssef, G.C., Huang, R., Li, G., Kim, J., Lee, E.Q., Nayak, L., Chukwueke, U., Beroukhim, R., et al. (2024). Neoadjuvant anti-PD1 immunotherapy for surgically accessible recurrent glioblastoma: clinical and molecular outcomes of a stage 2 single-arm expansion cohort. Nat. Commun. *15*, 10757. https://doi.org/10.1038/s41467-024-54326-7.

30. Long, G.V., Shklovskaya, E., Satgunaseelan, L., Mao, Y., da Silva, I.P., Perry, K.A., Diefenbach, R.J., Gide, T.N., Shivalingam, B., Buckland, M.E., et al. (2025). Neoadjuvant triplet immune checkpoint blockade in newly diagnosed glioblastoma. Nat. Med. *31*, 1557–1566. https://doi.org/10.1038/s41591-025-03512-1.

31. Trouillas, P., and Lapras, C. (1970). [Active immunotherapy of cerebral tumor. 20 cases]. Neurochirurgie *16*, 143–170.

32. Takakura, K., Miki, Y., and Kubo, O. (1975). Adjuvant immunotherapy for malignant brain tumors in infants and children. Childs Brain *1*, 141–147. https://doi.org/10.1159/000119563.

33. Young, H., Kaplan, A., and Regelson, W. (1977). Immunotherapy with autologous white cell infusions ("lymphocytes") in the treatment of recurrrent glioblastoma multiforme: a preliminary report. Cancer *40*, 1037–1044. https://doi.org/10.1002/1097-0142(197709)40:3<1037::aid-cncr2820400311>3.0.co;2-9.

34. Neuwelt, E.A., Clark, K., Kirkpatrick, J.B., and Toben, H. (1978). Clinical studies of intrathecal autologous lymphocyte infusions in patients with malignant glioma: a toxicity study. Ann. Neurol. *4*, 307–312. https://doi.org/10.1002/ana.410040404.

35. Mita, R., and Iwabuchi, T. (1979). Treatment of malignant glioma by immunotherapy using lymphocytes sensitized with tumor-specific immune RNA (author's transl)]. Neurol. Med.-Chir. *19*, 335–341. https://doi.org/10.2176/nmc.19.335.

36. Ishizawa, A. (1981). [Immunotherapy for malignant gliomas (author's transl)]. Neurol. Med.-Chir. *21*, 179–191. https://doi.org/10.2176/nmc.21.179.

37. Steinbok, P., Thomas, J.P., Grossman, L., and Dolman, C.L. (1984). Intratumoral autologous mononuclear cells in the treatment of recurrent glioblastoma multiforme. A phase 1 (toxicity) study. J. Neuro Oncol. *2*, 147–151. https://doi.org/10.1007/bf00177901.

38. Vaquero, J., Martínez, R., Barbolla, L., de Haro, J., de Oya, S., Coca, S., and Ramiro, J. (1987). Intrathecal injection of autologous leucocytes in glioblastoma: circulatory dynamics within the subarachnoid space and clinical results. Acta Neurochir. *89*, 37–42. https://doi.org/10.1007/bf01406665.

39. Ingram, M., Jacques, S., Freshwater, D.B., Techy, G.B., Shelden, C.H., and Helsper, J.T. (1987). Salvage immunotherapy of malignant glioma. Arch. Surg. *122*, 1483–1486. https://doi.org/10.1001/archsurg.1987.01400240131025.

40. Ingram, M., Shelden, C.H., Jacques, S., Skillen, R.G., Bradley, W.G., Techy, G.B., Freshwater, D.B., Abts, R.M., and Rand, R.W. (1987). Preliminary clinical trial of immunotherapy for malignant glioma. J. Biol. Response Mod. *6*, 489–498.

41. Ingram, M., Buckwalter, J.G., Jacques, D.B., Freshwater, D.B., Abts, R.M., Techy, G.B., Miyagi, K., Shelden, C.H., Rand, R.W., and English, L.W. (1990). Immunotherapy for recurrent malignant glioma: an interim report on survival. Neurol. Res. *12*, 265–273. https://doi.org/10.1080/01616412.1990.11739955.

42. Vaquero, J., Martínez, R., Oya, S., Coca, S., Barbolla, L., Ramiro, J., and Salazar, F.G. (1989). Intratumoural injection of autologous lymphocytes plus human lymphoblastoid interferon for the treatment of glioblastoma. Acta Neurochir. *98*, 35–41. https://doi.org/10.1007/bf01407174.

43. Vaquero, J., Martínez, R., Ramiro, J., Salazar, F.G., Barbolla, L., and Regidor, C. (1991). Immunotherapy of glioblastoma with intratumoural administration of autologous lymphocytes and human lymphoblastoid interferon. A further clinical study. Acta Neurochir. *109*, 42–45. https://doi.org/10.1007/bf01405696.

44. Moriki, A. (1989). [Induction of LAK cells and CTL in patients with brain tumor and research of its clinical application]. Nihon Geka Hokan *58*, 107–118.

45. Hirotsu, T., Mineta, T., Ichinose, M., Toda, K., Fukuyama, K., and Tabuchi, K. (2001). Adoptive immunotherapy for malignant brain tumors using human peripheral blood mononuclear cells activated by the Streptococcal preparation OK-432. Neurol. Med.-Chir. *41*, 387–392. https://doi.org/10.2176/nmc.41.387.

46. Lotze, M.T., Grimm, E.A., Mazumder, A., Strausser, J.L., and Rosenberg, S.A. (1981). Lysis of fresh and cultured autologous tumor by human lymphocytes cultured in T-cell growth factor. Cancer Res. *41*, 4420–4425.

47. Grimm, E.A., Mazumder, A., Zhang, H.Z., and Rosenberg, S.A. (1982). Lymphokine-activated killer cell phenomenon. Lysis of natural killer-resistant fresh solid tumor cells by interleukin 2-activated autologous human peripheral blood lymphocytes. J. Exp. Med. *155*, 1823–1841. https://doi.org/10.1084/jem.155.6.1823.

48. Mulé, J.J., Shu, S., Schwarz, S.L., and Rosenberg, S.A. (1984). Adoptive immunotherapy of established pulmonary metastases with LAK cells and recombinant interleukin-2. Science *225*, 1487–1489. https://doi.org/10.1126/science.6332379.

49. Rosenberg, S.A., Lotze, M.T., Muul, L.M., Leitman, S., Chang, A.E., Ettinghausen, S.E., Matory, Y.L., Skibber, J.M., Shiloni, E., Vetto, J.T., et al. (1985). Observations on the systemic administration of autologous lymphokine-activated killer cells and recombinant interleukin-2 to patients with metastatic cancer. N. Engl. J. Med. *313*, 1485–1492. https://doi.org/10.1056/nejm198512053132327.

50. Jacobs, S.K., Wilson, D.J., Kornblith, P.L., and Grimm, E.A. (1986). Interleukin-2 and autologous lymphokine-activated killer cells in the treatment of malignant glioma. Preliminary report. J. Neurosurg. *64*, 743–749. https://doi.org/10.3171/jns.1986.64.5.0743.

51. Silvani, A., Salmaggi, A., Parmiani, G., and Boiardi, A. (1994). Successful adoptive immunotherapy with lymphokine-activated killer cells in the treatment of medulloblastoma disseminated via cerebrospinal fluid: case report. Neurosurgery *34*, 1078–1081. https://doi.org/10.1227/00006123-199406000-00021.

52. Itoh, K., Sawamura, Y., Hosokawa, M., and Kobayashi, H. (1988). Scintigraphy with In-111 labeled lymphokine-activated killer cells of malignant brain tumor. Radiat. Med. *6*, 276–281.

53. Hayes, R.L., Koslow, M., Hiesiger, E.M., Hymes, K.B., Hochster, H.S., Moore, E.J., Pierz, D.M., Chen, D.K., Budzilovich, G.N., and Ransohoff, J. (1995). Improved long term survival after intracavitary interleukin-2 and lymphokine-activated killer cells for adults with recurrent malignant glioma. Cancer *76*, 840–852. https://doi.org/10.1002/1097-0142(19950901)76:5<840::aid-cncr2820760519>3.0.co;2-r.

54. Shimizu, K., Okamoto, Y., Miyao, Y., Yamada, M., Ushio, Y., Hayakawa, T., Ikeda, H., and Mogami, H. (1987). Adoptive immunotherapy of human meningeal gliomatosis and carcinomatosis with LAK cells and recombinant interleukin-2. J. Neurosurg. *66*, 519–521. https://doi.org/10.3171/jns.1987.66.4.0519.

55. Yoshida, S., Takai, N., Saito, T., and Tanaka, R. (1987). Adoptive immunotherapy in patients with malignant glioma]. Gan To Kagaku Ryoho. *14*, 1930–1932.

56. Merchant, R.E., Grant, A.J., Merchant, L.H., and Young, H.F. (1988). Adoptive immunotherapy for recurrent glioblastoma multiforme using lymphokine activated killer cells and recombinant interleukin-2. Cancer *62*, 665–671. https://doi.org/10.1002/1097-0142(19880815)62:4<665::aid-cncr2820620403>3.0.co;2-o.

57. Merchant, R.E., Merchant, L.H., Cook, S.H., McVicar, D.W., and Young, H.F. (1988). Intralesional infusion of lymphokine-activated killer (LAK) cells and recombinant interleukin-2 (rIL-2) for the treatment of patients with malignant brain tumor. Neurosurgery *23*, 725–732. https://doi.org/10.1227/00006123-198812000-00007.

58. Yoshida, S., Takai, N., Ono, K., Saito, T., and Tanaka, R. (1988). Observations on the local administration of autologous lymphokine activated killer cells and recombinant interleukin-2 in patients with malignant gliomas]. No Shinkei *40*, 119–125.

59. Yoshida, S., Tanaka, R., Takai, N., and Ono, K. (1988). Local administration of autologous lymphokine-activated killer cells and recombinant interleukin 2 to patients with malignant brain tumors. Cancer Res. *48*, 5011–5016.

60. Atkinson, L.L., Merchant, R.E., Ghatak, N.R., and Young, H.F. (1989). Sterile abscesses in glioma patients treated by intraparenchymal injection of lymphokine-activated killer cells and recombinant interleukin-2: case reports. Neurosurgery *25*, 805–810. https://doi.org/10.1097/00006123-198911000-00019.

61. Barba, D., Saris, S.C., Holder, C., Rosenberg, S.A., and Oldfield, E.H. (1989). Intratumoral LAK cell and interleukin-2 therapy of human gliomas. J. Neurosurg. *70*, 175–182. https://doi.org/10.3171/jns.1989.70.2.0175.

62. Naganuma, H., Kimurat, R., Sasaki, A., Fukamachi, A., Nukui, H., and Tasaka, K. (1989). Complete remission of recurrent glioblastoma multiforme following local infusions of lymphokine activated killer cells. Case report. Acta Neurochir. *99*, 157–160. https://doi.org/10.1007/bf01402326.

63. Shimizu, K., Tamura, K., Yamada, M., Okamoto, Y., Miyao, Y., Park, K., Matsui, Y., Hayakawa, T., Takimoto, H., and Mogami, H. (1989). Adoptive immunotherapy in patients with medulloblastoma by LAK cells]. No Shinkei *41*, 991–995.

64. Munari, L., Silvani, A., Passerini, C.G., Radrizzani, M., Parmiani, G., and Boiardi, A. (1990). Adoptive immunotherapy with adherent lymphokine-activated killer (A-LAK) cells in glioblastoma multiforme. J. Neurosurg. Sci. *34*, 283–288.

65. Nitta, T., Ishizawa, A., Ito, M., Sato, K., Yagita, H., and Kumura, K. (1990). [Induction of cytotoxicity from human lymphocytes coated with bispecific antibody against human glioma cells]. Noshinkeigeka *18*, 1001–1006.

66. Nitta, T., Sato, K., Yagita, H., Okumura, K., and Ishii, S. (1990). Preliminary trial of specific targeting therapy against malignant glioma. Lancet *335*, 368–371. https://doi.org/10.1016/0140-6736(90)90205-j.

67. Lillehei, K.O., Mitchell, D.H., Johnson, S.D., McCleary, E.L., and Kruse, C.A. (1991). Long-term follow-up of patients with recurrent malignant gliomas treated with adjuvant adoptive immunotherapy. Neurosurgery *28*, 16–23. https://doi.org/10.1097/00006123-199101000-00003.

68. Thomas, C., Schober, R., Lenard, H.G., Lumenta, C.B., Jacques, D.B., and Wechsler, W. (1992). Immunotherapy with stimulated autologous lymphocytes in a case of a juvenile anaplastic glioma. Neuropediatrics *23*, 123–125. https://doi.org/10.1055/s-2008-1071326.

69. Blancher, A., Roubinet, F., Grancher, A.S., Tremoulet, M., Bonaté, A., Delisle, M.B., Calot, J.P., Pourreau, C., Franks, C., Ducos, J., et al. (1993). Local immunotherapy of recurrent glioblastoma multiforme by intracerebral perfusion of interleukin-2 and LAK cells. Eur. Cytokine Netw. *4*, 331–341.

70. Ibayashi, Y., Yamaki, T., Kawahara, T., Daibo, M., Kubota, T., Uede, T., Tanabe, S., and Hashi, K. (1993). Effect of local administration of lymphokine-activated killer cells and interleukin-2 on malignant brain tumor patients. Neurol. Med.-Chir. *33*, 448–457. https://doi.org/10.2176/nmc.33.448.

71. Jeffes, E.W., 3rd, Beamer, Y.B., Jacques, S., Silberman, R.S., Vayuvegula, B., Gupta, S., Coss, J.S., Yamamoto, R.S., and Granger, G.A. (1993). Therapy of recurrent high grade gliomas with surgery, and

autologous mitogen activated IL-2 stimulated killer (MAK) lymphocytes: I. Enhancement of MAK lytic activity and cytokine production by PHA and clinical use of PHA. J. Neuro Oncol. *15*, 141–155. https://doi.org/10.1007/bf01053935.

72. Nagane, M., Oyama, H., Shibui, S., and Nomura, K. (1993). Recurrence with tumor bleeding in a patient with malignant astrocytoma during the treatment with intracranial injection of lymphokine-activated killer cells–a case report. No To Shinkei *45*, 547–551.

73. Boiardi, A., Silvani, A., Ruffini, P.A., Rivoltini, L., Parmiani, G., Broggi, G., and Salmaggi, A. (1994). Loco-regional immunotherapy with recombinant interleukin-2 and adherent lymphokine-activated killer cells (A-LAK) in recurrent glioblastoma patients. Cancer Immunol. Immunother. *39*, 193–197. https://doi.org/10.1007/bf01533386.

74. Nakagawa, K., Kamezaki, T., Shibata, Y., Tsunoda, T., Meguro, K., and Nose, T. (1995). Effect of lymphokine-activated killer cells with or without radiation therapy against malignant brain tumors. Neurol. Med.-Chir. *35*, 22–27. https://doi.org/10.2176/nmc.35.22.

75. Sankhla, S.K., Nadkarni, J.S., and Bhagwati, S.N. (1996). Adoptive immunotherapy using lymphokine-activated killer (LAK) cells and interleukin-2 for recurrent malignant primary brain tumors. J. Neuro Oncol. *27*, 133–140. https://doi.org/10.1007/bf00177476.

76. Naganuma, H., Sasaki, A., Satoh, E., Nagasaka, M., Isoe, S., Nakano, S., and Nukui, H. (1997). Long-term survival in a young patient with anaplastic glioma. Brain Tumor Pathol. *14*, 71–74. https://doi.org/10.1007/bf02478872.

77. Hayes, R.L., Arbit, E., Odaimi, M., Pannullo, S., Scheff, R., Kravchinskiy, D., and Zaroulis, C. (2001). Adoptive cellular immunotherapy for the treatment of malignant gliomas. Crit. Rev. Oncol. Hematol. *39*, 31–42. https://doi.org/10.1016/s1040-8428(01)00122-6.

78. Huang, Y., Hayes, R.L., Wertheim, S., Arbit, E., and Scheff, R. (2001). Treatment of refractory recurrent malignant glioma with adoptive cellular immunotherapy: a case report. Crit. Rev. Oncol. Hematol. *39*, 17–23. https://doi.org/10.1016/s1040-8428(01)00120-2.

79. Dillman, R.O., Duma, C.M., Schiltz, P.M., DePriest, C., Ellis, R.A., Okamoto, K., Beutel, L.D., De Leon, C., and Chico, S. (2004). Intracavitary placement of autologous lymphokine-activated killer (LAK) cells after resection of recurrent glioblastoma. J. Immunother. *27*, 398–404. https://doi.org/10.1097/00002371-200409000-00009.

80. Dillman, R.O., Duma, C.M., Ellis, R.A., Cornforth, A.N., Schiltz, P.M., Sharp, S.L., and DePriest, M.C. (2009). Intralesional lymphokine-activated killer cells as adjuvant therapy for primary glioblastoma. J. Immunother. *32*, 914–919. https://doi.org/10.1097/CJI.0b013e3181b2910f.

81. Okamoto, Y., Shimizu, K., Tamura, K., Miyao, Y., Yamada, M., Matsui, Y., Tsuda, N., Takimoto, H., Hayakawa, T., and Mogami, H. (1988). An adoptive immunotherapy of patients with medulloblastoma by lymphokine-activated killer cells (LAK). Acta Neurochir. *94*, 47–52. https://doi.org/10.1007/bf01406615.

82. Hida, T., Koike, K., Sekido, Y., Nishida, K., Sugiura, T., Ariyoshi, Y., Takahashi, T., and Ueda, R. (1991). Epitope analysis of cluster 1 and NK cell-related monoclonal antibodies. Br. J. Cancer Suppl. *14*, 24–28.

83. Rosenberg, S.A., Spiess, P., and Lafreniere, R. (1986). A new approach to the adoptive immunotherapy of cancer with tumor-infiltrating lymphocytes. Science *233*, 1318–1321. https://doi.org/10.1126/science.3489291.

84. Smith, M.M., Thompson, J.E., Castillo, M., Cush, S., Mukherji, S.K., Miller, C.H., and Quattrocchi, K.B. (1996). MR of recurrent high-grade astrocytomas after intralesional immunotherapy. AJNR. Am. J. Neuroradiol. *17*, 1065–1071.

85. Holladay, F.P., Heitz-Turner, T., Bayer, W.L., and Wood, G.W. (1996). Autologous tumor cell vaccination combined with adoptive cellular immunotherapy in patients with grade III/IV astrocytoma. J. Neuro Oncol. *27*, 179–189. https://doi.org/10.1007/bf00177482.

86. Kruse, C.A., Cepeda, L., Owens, B., Johnson, S.D., Stears, J., and Lillehei, K.O. (1997). Treatment of recurrent glioma with intracavitary alloreactive cytotoxic T lymphocytes and interleukin-2. Cancer Immunol. Immunother. *45*, 77–87. https://doi.org/10.1007/s002620050405.

87. Plautz, G.E., Barnett, G.H., Miller, D.W., Cohen, B.H., Prayson, R.A., Krauss, J.C., Luciano, M., Kangisser, D.B., and Shu, S. (1998). Systemic T cell adoptive immunotherapy of malignant gliomas. J. Neurosurg. *89*, 42–51. https://doi.org/10.3171/jns.1998.89.1.0042.

88. Tsurushima, H., Liu, S.Q., Tuboi, K., Matsumura, A., Yoshii, Y., Nose, T., Saijo, K., and Ohno, T. (1999). Reduction of end-stage malignant glioma by injection with autologous cytotoxic T lymphocytes. Jpn. J. Cancer Res. *90*, 536–545. https://doi.org/10.1111/j.1349-7006.1999.tb00781.x.

89. Plautz, G.E., Miller, D.W., Barnett, G.H., Stevens, G.H., Maffett, S., Kim, J., Cohen, P.A., and Shu, S. (2000). T cell adoptive immunotherapy of newly diagnosed gliomas. Clin. Cancer Res. *6*, 2209–2218.

90. Wood, G.W., Holladay, F.P., Turner, T., Wang, Y.Y., and Chiga, M. (2000). A pilot study of autologous cancer cell vaccination and cellular immunotherapy using anti-CD3 stimulated lymphocytes in patients with recurrent grade III/IV astrocytoma. J. Neuro Oncol. *48*, 113–120. https://doi.org/10.1023/a:1006456421177.

91. Sloan, A.E., Dansey, R., Zamorano, L., Barger, G., Hamm, C., Diaz, F., Baynes, R., and Wood, G. (2000). Adoptive immunotherapy in patients with recurrent malignant glioma: preliminary results of using autologous whole-tumor vaccine plus granulocyte-macrophage colony-stimulating factor and adoptive transfer of anti-CD3-activated lymphocytes. Neurosurg. Focus *9*, e9. https://doi.org/10.3171/foc.2000.9.6.10.

92. Tsuboi, K., Saijo, K., Ishikawa, E., Tsurushima, H., Takano, S., Morishita, Y., and Ohno, T. (2003). Effects of local injection of ex vivo expanded autologous tumor-specific T lymphocytes in cases with recurrent malignant gliomas. Clin. Cancer Res. *9*, 3294–3302.

93. Peres, E., Wood, G.W., Poulik, J., Baynes, R., Sood, S., Abidi, M.H., Klein, J., Bhambhani, K., Dansey, R., and Abella, E. (2008). High-dose chemotherapy and adoptive immunotherapy in the treatment of recurrent pediatric brain tumors. Neuropediatrics *39*, 151–156. https://doi.org/10.1055/s-0028-1093333.

94. Katakura, R., Suzuki, Y., Sekine, T., Sasaki, Y.F., and Fujimiya, Y. (2010). Therapeutic Efficacy of Adoptive Cell Transfer on Survival of Patients with Glioblastoma Multiforme: Case Reports. Case Rep. Oncol. *3*, 110–124. https://doi.org/10.1159/000313629.

95. Crough, T., Beagley, L., Smith, C., Jones, L., Walker, D.G., and Khanna, R. (2012). Ex vivo functional analysis, expansion and adoptive transfer of cytomegalovirus-specific T-cells in patients with glioblastoma multiforme. Immunol. Cell Biol. *90*, 872–880. https://doi.org/10.1038/icb.2012.19.

96. Kanemura, Y., Sumida, M., Okita, Y., Yoshioka, E., Yamamoto, A., Kanematsu, D., Handa, Y., Fukusumi, H., Inazawa, Y., Takada, A.I., et al. (2017). Systemic Intravenous Adoptive Transfer of Autologous Lymphokine-activated $\alpha\beta$ T-Cells Improves Temozolomide-induced Lymphopenia in Patients with Glioma. Anticancer Res. *37*, 3921–3932. https://doi.org/10.21873/anticanres.11775.

97. Kong, D.S., Nam, D.H., Kang, S.H., Lee, J.W., Chang, J.H., Kim, J.H., Lim, Y.J., Koh, Y.C., Chung, Y.G., Kim, J.M., and Kim, C.H. (2017). Phase III randomized trial of autologous cytokine-induced killer cell immunotherapy for newly diagnosed glioblastoma in Korea. Oncotarget *8*, 7003–7013. https://doi.org/10.18632/oncotarget.12273.

98. Kirkin, A.F., Dzhandzhugazyan, K.N., Guldberg, P., Fang, J.J., Andersen, R.S., Dahl, C., Mortensen, J., Lundby, T., Wagner, A., Law, I., et al. (2018). Adoptive cancer immunotherapy using DNA-demethylated T helper cells as antigen-presenting cells. Nat. Commun. *9*, 785. https://doi.org/10.1038/s41467-018-03217-9.

99. Roszman, T., Elliott, L., and Brooks, W. (1991). Modulation of T-cell function by gliomas. Immunol. Today *12*, 370–374. https://doi.org/10.1016/0167-5699(91)90068-5.

100. Zinkernagel, R.M., and Doherty, P.C. (1974). Restriction of in vitro T cell-mediated cytotoxicity in lymphocytic choriomeningitis within a syngeneic

or semiallogeneic system. Nature *248*, 701–702. https://doi.org/10.1038/248701a0.

101. Facoetti, A., Nano, R., Zelini, P., Morbini, P., Benericetti, E., Ceroni, M., Campoli, M., and Ferrone, S. (2005). Human leukocyte antigen and antigen processing machinery component defects in astrocytic tumors. Clin. Cancer Res. *11*, 8304–8311. https://doi.org/10.1158/1078-0432.Ccr-04-2588.

102. Khatua, S., Cooper, L.J.N., Sandberg, D.I., Ketonen, L., Johnson, J.M., Rytting, M.E., Liu, D.D., Meador, H., Trikha, P., Nakkula, R.J., et al. (2020). Phase I study of intraventricular infusions of autologous ex vivo expanded NK cells in children with recurrent medulloblastoma and ependymoma. Neuro Oncol. *22*, 1214–1225. https://doi.org/10.1093/neuonc/noaa047.

103. Lim, J., Park, Y., Ahn, J.W., Sim, J., Kang, S.J., Hwang, S., Chun, J., Choi, H., Kim, S.H., Chun, D.H., et al. (2021). Autologous adoptive immune-cell therapy elicited a durable response with enhanced immune reaction signatures in patients with recurrent glioblastoma: An open label, phase I/IIa trial. PLoS One *16*, e0247293. https://doi.org/10.1371/journal.pone.0247293.

104. Asl, N.S., Behfar, M., Amiri, R.S., Mohseni, R., Azimi, M., Firouzi, J., Faranoush, M., Izadpanah, A., Mohmmad, M., Hamidieh, A.A., et al. (2023). Intra-lesion injection of activated Natural Killer (NK) cells in recurrent malignant brain tumors. Int. Immunopharmacol. *120*, 110345. https://doi.org/10.1016/j.intimp.2023.110345.

105. Burger, M.C., Forster, M.T., Romanski, A., Straßheimer, F., Macas, J., Zeiner, P.S., Steidl, E., Herkt, S., Weber, K.J., Schupp, J., et al. (2023). Intracranial injection of natural killer cells engineered with a HER2-targeted chimeric antigen receptor in patients with recurrent glioblastoma. Neuro Oncol. *25*, 2058–2071. https://doi.org/10.1093/neuonc/noad087.

106. Mahdizadeh, H., Izadpanah, A., Nouri, Y., Shams, P., Daneshjou, D., Ahari, A.A., Tabibkhooei, A., Haghighatkhah, H., Vosough, M., Faranoush, P., et al. (2025). The safety and feasibility of multiple intrathecal injections of allogenic NK cells in pediatrics with refractory/recurrent brain tumors. BMC Cancer *25*, 952. https://doi.org/10.1186/s12885-025-14314-6.

107. Carlsten, M., and Childs, R.W. (2015). Genetic Manipulation of NK Cells for Cancer Immunotherapy: Techniques and Clinical Implications. Front. Immunol. *6*, 266. https://doi.org/10.3389/fimmu.2015.00266.

108. Eshhar, Z., Waks, T., Gross, G., and Schindler, D.G. (1993). Specific activation and targeting of cytotoxic lymphocytes through chimeric single chains consisting of antibody-binding domains and the gamma or zeta subunits of the immunoglobulin and T-cell receptors. Proc. Natl. Acad. Sci. USA *90*, 720–724. https://doi.org/10.1073/pnas.90.2.720.

109. Brocker, T., Peter, A., Traunecker, A., and Karjalainen, K. (1993). New simplified molecular design for functional T cell receptor. Eur. J. Immunol. *23*, 1435–1439. https://doi.org/10.1002/eji.1830230705.

110. Yarmarkovich, M., Marshall, Q.F., Warrington, J.M., Premaratne, R., Farrel, A., Groff, D., Li, W., di Marco, M., Runbeck, E., Truong, H., et al. (2023). Targeting of intracellular oncoproteins with peptide-centric CARs. Nature *623*, 820–827. https://doi.org/10.1038/s41586-023-06706-0.

111. Fielding, A.K., Richards, S.M., Chopra, R., Lazarus, H.M., Litzow, M.R., Buck, G., Durrant, I.J., Luger, S.M., Marks, D.I., Franklin, I.M., et al. (2007). Outcome of 609 adults after relapse of acute lymphoblastic leukemia (ALL); an MRC UKALL12/ECOG 2993 study. Blood *109*, 944–950. https://doi.org/10.1182/blood-2006-05-018192.

112. Vitanza, N.A., Ronsley, R., Choe, M., Seidel, K., Huang, W., Rawlings-Rhea, S.D., Beam, M., Steinmetzer, L., Wilson, A.L., Brown, C., et al. (2025). Intracerebroventricular B7-H3-targeting CAR T cells for diffuse intrinsic pontine glioma: a phase 1 trial. Nat. Med. *31*, 861–868. https://doi.org/10.1038/s41591-024-03451-3.

113. Del Bufalo, F., De Angelis, B., Caruana, I., Del Baldo, G., De Ioris, M.A., Serra, A., Mastronuzzi, A., Cefalo, M.G., Pagliara, D., Amicucci, M., et al. (2023). GD2-CART01 for Relapsed or Refractory High-Risk Neuroblastoma. N. Engl. J. Med. *388*, 1284–1295. https://doi.org/10.1056/NEJMoa2210859.

114. Ventin, M., Cattaneo, G., Maggs, L., Arya, S., Wang, X., and Ferrone, C.R. (2024). Implications of High Tumor Burden on Chimeric Antigen Receptor T-Cell Immunotherapy: A Review. JAMA Oncol. *10*, 115–121. https://doi.org/10.1001/jamaoncol.2023.4504.

115. Timpanaro, A., Song, E.Z., Amwas, N., Chiu, C.H., Ronsley, R., Taylor, M.R., Foster, J.B., Wang, L.D., and Vitanza, N.A. (2025). Evolving CAR T-Cell Therapy to Overcome the Barriers in Treating Pediatric Central Nervous System Tumors. Cancer Discov. *15*, 890–902. https://doi.org/10.1158/2159-8290.Cd-24-1465.

116. Burns, I., Gwynne, W.D., Suk, Y., Custers, S., Chaudhry, I., Venugopal, C., and Singh, S.K. (2022). The Road to CAR T-Cell Therapies for Pediatric CNS Tumors: Obstacles and New Avenues. Front. Oncol. *12*, 815726. https://doi.org/10.3389/fonc.2022.815726.

117. Haydar, D., Ibañez-Vega, J., and Krenciute, G. (2021). T-Cell Immunotherapy for Pediatric High-Grade Gliomas: New Insights to Overcoming Therapeutic Challenges. Front. Oncol. *11*, 718030. https://doi.org/10.3389/fonc.2021.718030.

118. Park, S., Maus, M.V., and Choi, B.D. (2024). CAR-T cell therapy for the treatment of adult high-grade gliomas. npj Precis. Oncol. *8*, 279. https://doi.org/10.1038/s41698-024-00753-0.

119. Ronsley, R., Bertrand, K.C., Song, E.Z., Timpanaro, A., Choe, M., Tlais, D., Vitanza, N.A., and Park, J.R. (2024). CAR T cell therapy for pediatric central nervous system tumors: a review of the literature and current North American trials. Cancer Metastasis Rev. *43*, 1205–1216. https://doi.org/10.1007/s10555-024-10208-4.

120. Yaacoub, S., Vannoy, E., Maslova, S., Haffey, A., Khorsandi, K., Sheybani, N., and Haydar, D. (2025). CAR-T cell therapy in brain malignancies: obstacles in the face of cellular trafficking and persistence. Front. Immunol. *16*, 1596499. https://doi.org/10.3389/fimmu.2025.1596499.

121. Begley, S.L., O'Rourke, D.M., and Binder, Z.A. (2025). CAR T cell therapy for glioblastoma: A review of the first decade of clinical trials. Mol. Ther. *33*, 2454–2461. https://doi.org/10.1016/j.ymthe.2025.03.004.

122. Zhou, D., Zhu, X., and Xiao, Y. (2024). Advances in CAR-T therapy for central nervous system tumors. Biomark. Res. *12*, 132. https://doi.org/10.1186/s40364-024-00679-6.

123. Binder, Z.A., Bagley, S.J., Foster, J.B., and O'Rourke, D.M. (2026). The development of CAR T cells for patients with CNS malignancies. Nat. Rev. Clin. Oncol. *23*, 137–150. https://doi.org/10.1038/s41571-025-01102-1.

124. Grewal, E.P., Nahed, B.V., Carter, B.S., Gerstner, E.R., Curry, W.T., Maus, M.V., and Choi, B.D. (2025). Clinical progress in the development of CAR T cells to treat malignant glioma. J. Neuro Oncol. *171*, 571–579. https://doi.org/10.1007/s11060-024-04909-7.

125. Królikowska, A., and Tarnowski, M. (2025). CAR-T cells immunotherapy in the treatment of glioblastoma. Cancer Immunol. Immunother. *74*, 363. https://doi.org/10.1007/s00262-025-04222-w.

126. Sabahi, M., Fathi Jouzdani, A., Sadeghian, Z., Dabbagh Ohadi, M.A., Sultan, H., Salehipour, A., Maniakhina, L., Rezaei, N., Adada, B., Mansouri, A., and Borghei-Razavi, H. (2025). CAR-engineered NK cells versus CAR T cells in treatment of glioblastoma; strength and flaws. J. Neuro Oncol. *171*, 495–530. https://doi.org/10.1007/s11060-024-04876-z.

127. Monje, M., Mahdi, J., Majzner, R., Yeom, K.W., Schultz, L.M., Richards, R.M., Barsan, V., Song, K.W., Kamens, J., Baggott, C., et al. (2025). Intravenous and intracranial GD2-CAR T cells for H3K27M(+) diffuse midline gliomas. Nature *637*, 708–715. https://doi.org/10.1038/s41586-024-08171-9.

128. Keu, K.V., Witney, T.H., Yaghoubi, S., Rosenberg, J., Kurien, A., Magnusson, R., Williams, J., Habte, F., Wagner, J.R., Forman, S., et al. (2017). Reporter gene imaging of targeted T cell immunotherapy in

CellPress
OPEN ACCESS

recurrent glioma. Sci. Transl. Med. *9*, eaag2196. https://doi.org/10.1126/scitranslmed.aag2196.

129. Brown, C.E., Rodriguez, A., Palmer, J., Ostberg, J.R., Naranjo, A., Wagner, J.R., Aguilar, B., Starr, R., Weng, L., Synold, T.W., et al. (2022). Off-the-shelf, steroid-resistant, IL13Rα2-specific CAR T cells for treatment of glioblastoma. Neuro Oncol. *24*, 1318–1330. https://doi.org/10.1093/neuonc/noac024.

130. Vitanza, N.A., Wilson, A.L., Huang, W., Seidel, K., Brown, C., Gustafson, J.A., Yokoyama, J.K., Johnson, A.J., Baxter, B.A., Koning, R.W., et al. (2023). Intraventricular B7-H3 CAR T Cells for Diffuse Intrinsic Pontine Glioma: Preliminary First-in-Human Bioactivity and Safety. Cancer Discov. *13*, 114–131. https://doi.org/10.1158/2159-8290.CD-22-0750.

131. Tang, X., Wang, Y., Huang, J., Zhang, Z., Liu, F., Xu, J., Guo, G., Wang, W., Tong, A., and Zhou, L. (2021). Administration of B7-H3 targeted chimeric antigen receptor-T cells induce regression of glioblastoma. Signal Transduct. Target. Ther. *6*, 125. https://doi.org/10.1038/s41392-021-00505-7.

132. Gust, J., Cole, B.L., Ronsley, R., Wilson, A.L., Seidel, K., Wendler, J., Pattabhi, S., Brown, C., Rawlings-Rhea, S.D., Shtanukhina, N., et al. (2025). Locoregional Infusion of EGFR806-CAR T Cells for Recurrent or Refractory Pediatric CNS Tumors: Results of the Completed BrainChild02 Phase 1 Clinical Trial. Neuro Oncol. *27*, 2170–2181. https://doi.org/10.1093/neuonc/noaf064.

133. O'Rourke, D.M., Nasrallah, M.P., Desai, A., Melenhorst, J.J., Mansfield, K., Morrissette, J.J.D., Martinez-Lage, M., Brem, S., Maloney, E., Shen, A., et al. (2017). A single dose of peripherally infused EGFRvIII-directed CAR T cells mediates antigen loss and induces adaptive resistance in patients with recurrent glioblastoma. Sci. Transl. Med. *9*, eaaa0984. https://doi.org/10.1126/scitranslmed.aaa0984.

134. Durgin, J.S., Henderson, F., Jr., Nasrallah, M.P., Mohan, S., Wang, S., Lacey, S.F., Melenhorst, J.J., Desai, A.S., Lee, J.Y.K., Maus, M.V., et al. (2021). Case Report: Prolonged Survival Following EGFRvIII CAR T Cell Treatment for Recurrent Glioblastoma. Front. Oncol. *11*, 669071. https://doi.org/10.3389/fonc.2021.669071.

135. Goff, S.L., Morgan, R.A., Yang, J.C., Sherry, R.M., Robbins, P.F., Restifo, N.P., Feldman, S.A., Lu, Y.C., Lu, L., Zheng, Z., et al. (2019). Pilot Trial of Adoptive Transfer of Chimeric Antigen Receptor-transduced T Cells Targeting EGFRvIII in Patients With Glioblastoma. J. Immunother. *42*, 126–135. https://doi.org/10.1097/cji.0000000000000260.

136. Bagley, S.J., Binder, Z.A., Lamrani, L., Marinari, E., Desai, A.S., Nasrallah, M.P., Maloney, E., Brem, S., Lustig, R.A., Kurtz, G., et al. (2024). Repeated peripheral infusions of anti-EGFRvIII CAR T cells in combination with pembrolizumab show no efficacy in glioblastoma: a phase 1 trial. Nat. Cancer *5*, 517–531. https://doi.org/10.1038/s43018-023-00709-6.

137. Lin, Q., Ba, T., Ho, J., Chen, D., Cheng, Y., Wang, L., Xu, G., Xu, L., Zhou, Y., Wei, Y., et al. (2021). First-in-Human Trial of EphA2-Redirected CAR T-Cells in Patients With Recurrent Glioblastoma: A Preliminary Report of Three Cases at the Starting Dose. Front. Oncol. *11*, 694941. https://doi.org/10.3389/fonc.2021.694941.

138. Liu, Z., Zhou, J., Yang, X., Liu, Y., Zou, C., Lv, W., Chen, C., Cheng, K.K.Y., Chen, T., Chang, L.J., et al. (2023). Safety and antitumor activity of GD2-Specific 4SCAR-T cells in patients with glioblastoma. Mol. Cancer *22*, 3. https://doi.org/10.1186/s12943-022-01711-9.

139. Vitanza, N.A., Johnson, A.J., Wilson, A.L., Brown, C., Yokoyama, J.K., Künkele, A., Chang, C.A., Rawlings-Rhea, S., Huang, W., Seidel, K., et al. (2021). Locoregional infusion of HER2-specific CAR T cells in children and young adults with recurrent or refractory CNS tumors: an interim analysis. Nat. Med. *27*, 1544–1552. https://doi.org/10.1038/s41591-021-01404-8.

140. Brown, C.E., Hibbard, J.C., Alizadeh, D., Blanchard, M.S., Natri, H.M., Wang, D., Ostberg, J.R., Aguilar, B., Wagner, J.R., Paul, J.A., et al. (2024). Locoregional delivery of IL-13Rα2-targeting CAR-T cells in recurrent high-grade glioma: a phase 1 trial. Nat. Med. *30*, 1001–1012. https://doi.org/10.1038/s41591-024-02875-1.

141. Barish, M.E., Aftabizadeh, M., Hibbard, J., Blanchard, M.S., Ostberg, J.R., Wagner, J.R., Manchanda, M., Paul, J., Stiller, T., Aguilar, B., et al. (2025). Chlorotoxin-directed CAR T cell therapy for recurrent glioblastoma: Interim clinical experience demonstrating feasibility and safety. Cell Rep. Med. *6*, 102302. https://doi.org/10.1016/j.xcrm.2025.102302.

142. Bagley, S.J., Desai, A.S., Fraietta, J.A., Silverbush, D., Chafamo, D., Freeburg, N.F., Gopikrishna, G.K., Rech, A.J., Nabavizadeh, A., Bagley, L.J., et al. (2025). Intracerebroventricular bivalent CAR T cells targeting EGFR and IL-13Rα2 in recurrent glioblastoma: a phase 1 trial. Nat. Med. *31*, 2778–2787. https://doi.org/10.1038/s41591-025-03745-0.

143. Brown, C.E., Aguilar, B., Starr, R., Yang, X., Chang, W.C., Weng, L., Chang, B., Sarkissian, A., Brito, A., Sanchez, J.F., et al. (2018). Optimization of IL13Rα2-Targeted Chimeric Antigen Receptor T Cells for Improved Anti-tumor Efficacy against Glioblastoma. Mol. Ther. *26*, 31–44. https://doi.org/10.1016/j.ymthe.2017.10.002.

144. Mahdi, J., Dietrich, J., Straathof, K., Roddie, C., Scott, B.J., Davidson, T.B., Prolo, L.M., Batchelor, T.T., Campen, C.J., Davis, K.L., et al. (2023). Tumor inflammation-associated neurotoxicity. Nat. Med. *29*, 803–810. https://doi.org/10.1038/s41591-023-02276-w.

145. Macdonald, D.R., Cascino, T.L., Schold, S.C., Jr., and Cairncross, J.G. (1990). Response criteria for phase II studies of supratentorial malignant glioma. J. Clin. Oncol. *8*, 1277–1280. https://doi.org/10.1200/jco.1990.8.7.1277.

146. Brandsma, D., Stalpers, L., Taal, W., Sminia, P., and van den Bent, M.J. (2008). Clinical features, mechanisms, and management of pseudoprogression in malignant gliomas. Lancet Oncol. *9*, 453–461. https://doi.org/10.1016/s1470-2045(08)70125-6.

147. Wen, P.Y., Macdonald, D.R., Reardon, D.A., Cloughesy, T.F., Sorensen, A.G., Galanis, E., Degroot, J., Wick, W., Gilbert, M.R., Lassman, A.B., et al. (2010). Updated response assessment criteria for high-grade gliomas: response assessment in neuro-oncology working group. J. Clin. Oncol. *28*, 1963–1972. https://doi.org/10.1200/jco.2009.26.3541.

148. Wen, P.Y., van den Bent, M., Youssef, G., Cloughesy, T.F., Ellingson, B.M., Weller, M., Galanis, E., Barboriak, D.P., de Groot, J., Gilbert, M.R., et al. (2023). RANO 2.0: Update to the Response Assessment in Neuro-Oncology Criteria for High- and Low-Grade Gliomas in Adults. J. Clin. Oncol. *41*, 5187–5199. https://doi.org/10.1200/jco.23.01059.

149. Brummer, A.B., Yang, X., Ma, E., Gutova, M., Brown, C.E., and Rockne, R.C. (2022). Dose-dependent thresholds of dexamethasone destabilize CAR T-cell treatment efficacy. PLoS Comput. Biol. *18*, e1009504. https://doi.org/10.1371/journal.pcbi.1009504.

Cell Reports Medicine, Volume *7*

Supplemental information

Cell therapy for brain tumors: The first 60 years

Sanya Mehta, Giedre Krenciute, and Stephen Gottschalk

Supplementary Table 1: Cell Therapy with lymphokine-activated killer cells

Year(s)	Pt(#)	Diagnosis	Treatment	Cell dose	Best clinical outcomes
1986[15,50]	10	Progressive glioma	auto LAKs and/or IL-2	$0.08\text{-}1\times10^{10}$ (ITu)	No benefit
1987[54]	1	Meningeal gliomatosis	auto LAKs and IL-2	$1\text{-}2\times10^{8}$ (ITu)	Improvement in symptoms for 7 mths
1987[55]<br>1988[58,59]	23	Recurrent anaplastic A	auto LAKs and IL-2	$1.2\text{-}3.24\times10^{8}$ (ITu)	1 pt: CR for >14 mths<br>1 pt: partial regression with no recurrence for >19 mths<br>2 pts: transient regression for 6-8 mths<br>7 pts: improvement in symptoms
1988[81]<br>1989[63]	6	MB with CSF dissemination	haplo LAKs and IL-2	$0.2\text{-}1\times10^{9}$ (ITu or IT)	1 pt: CR for >18 mths<br>3 pts: improvement in
1988[56]<br>1989[60]	13	Recurrent GBM	auto LAKs and IL-2	$0.1\text{-}5.8\times10^{9}$ (ITu)	1 pt: no recurrence for >12 mths
1988[57]<br>1989[60]	19	Primary or recurrent GBM or high-grade OG	auto LAKs and IL-2 during craniotomy	$0.1\text{-}1.5\times10^{10}$ (ITu)	8 pts: progression free for >6 mths
1989[61]	9	Progressive GBM and anaplastic A	auto LAKs and IL-2	$0.09\text{-}2.1\times10^{10}$ (ITu)	1 pt: PR for 9 mths, then SD for 4 mths
1989[62]<br>1997[76]	1	Recurrent anaplastic glioma	auto LAKs with IFN-β and ANCU	2.7×10^{9} (ITu)	CR for >9 years and 9 months
1990[65,66]	20	Primary GBM or anaplastic A	auto LAKs +/- bispecific antibody	$0.8\text{-}1.2\times10^{8}$ (ITu)	4 pts: CR for >8-18 mths<br>5 pts: partial regression for >8-18 mths
1990[64]<br>1994[73]	9	Recurrent GBM or anaplastic A	adherent auto LAKs and IL-2	1.5×10^{8} (ITu)	1 pt: CR for 28 wks<br>2 pts: PR for 25-28 wks<br>4 pts: SD for 12-36 wks
1991[67]	20	Recurrent GBM or anaplastic A	auto LAKs, stimulated lymphocytes, and IL-2	$0.19\text{-}2.75\times10^{10}$ (ITu)	No benefit
1992[68]	1	GBM or grade 3 A	auto LAKs and IL-2 during craniotomy, PHA-stimulated and tumor-sensitized lymphocytes	Unknown (ITu)	No benefit
1993[71]	19	Recurrent grade 3-4 HGG	auto LAKs and IL-2 during craniotomy +/- PHA coating of surgical cavity	$0.22\text{-}5.8\times10^{10}$ (ITu)	3 pts: transient regression for <4 mths<br>7 pts: Improvement in clinical symptoms for <4-8 mths
1993[72]	1	Recurrent grade 3 A	auto LAKs and IL-2	Unknown (ITu)	No benefit
1993[70]	8	Progressive GBM and anaplastic A	auto LAKs and IL-2	$0.55\text{-}9.37\times10^{8}$ (ITu)	1 pt: CR for >5 yrs<br>2 pt: PR for 6 and 11 mths
1993[69]	5	Recurrent GBM	auto LAKs and IL-2	Unknown (ITu)	No benefit
1994[51]	1	MB with CSF dissemination	auto LAKs and IL-2	7×10^{8} (IT)	CR for >30 mths
1995[74]	9	Primary or recurrent GBM or grade 3 A	auto LAKs and IL-2 +/- XRT	$3.7\text{-}9.49\times10^{7}$ (ITu)	2 pts: 2 CR for >20 and >90 mths<br>1 pt: PR for >24 mths<br>1 pt: transient PR
1995[53]<br>2001[77,78]	28	Primary or recurrent malignant glioma	auto LAKs and IL-2	$0.5\text{-}5\times10^{9}$ (ITu)	2 pts: CR for >3.4 yrs<br>1 pt: CR for >27 mths<br>2 pts: PR for 9.5 mths and 3.6 yrs<br>1 pt: SD for >3 yrs
1996[75]	10	Recurrent grade 2-3 A, GBM, AO, MB	auto LAKs	$1\text{-}2.5\times10^{6}$ (ITu)	2 pts: neurological improvements for 18 and 56 wks
2004[79]	40	Recurrent GBM	auto LAKs during craniotomy	$1\text{-}3\times10^{9}$ (ITu)	Improved survival compared to historical ctrls (avg: 17.5 vs 13.6 mths)
2009[80]	33	Primary GBM or grade 3 A following std primary therapy	auto LAKs during craniotomy	$0.93\text{-}2.57\times10^{9}$ (ITu)	Improved survival compared to historical ctrls (avg: 20.5 vs 12 mths)

Pt(#): Number of patients, *__Diagnosis column__:* A: astrocytoma, AO: astrocytoma-oligodendroglioma, CSF: cerebrospinal fluid, GBM: glioblastoma, HGG: high-grade glioma, MB: medulloblastoma, MM: metastatic meningioma, OA: oligoastrocytoma, OG: oligodendroglioma, std: standard*; __Treatment column__:* allo: allogeneic, ANCU: 1-(4-amino-2-methyl-5-pyrimidinyl) methyl-3-(2- chloroethyl)-3-nitrosourea hydrochloride, auto: autologous, haplo: haploidentical, IFN: interferon, IL: interleukin, lymphokine-activated killer cells: LAKs, PBMCs: peripheral blood mononuclear cells, PHA: phytohemagglutinin, XRT: radiation therapy*; __Cell dose column__:* IT: intrathecal, ITu: intratumoral*; __Best clinical outcome column__*: avg: average, CR: complete response, ctrls: controls, mths: months, PR: partial response, SD: stable disease, wks: weeks, yrs: years.

Supplementary Table 2: Cell Therapy with NK cells

Year(s)	Pt(#)	Diagnosis	Treatment	Cell dose	Best clinical outcomes
2004[23]	9	Recurrent malignant glioma	auto NK cells with IL-2 and IFN-β	$0.6\text{-}6.5x10^9$ (IV and ITu)	3 pts: PR<br>2 pts: MR
2020[102]	12	Recurrent MB and EPN	auto NK cells	$0.01\text{-}3x10^8/m^2$ (ICV)	1 pt: tumor regression for 3 wks<br>1pt: SD for 1 mth
2021[103]	14	Recurrent GBM	auto NK cells	$2\text{-}6x10^9$ (IV)	5 pts: tumor regressions for 28 – 76 mths
2023[104]	5	Recurrent GBM and grade 4 MB	haplo NK cells	$0.02\text{-}1x10^8$ (IT)	2 pts: SD for 40 days and 3 mths
2023[105]	9	Recurrent HER2+ GBM	irradiated HER2-CAR NK92 cell line	$0.1\text{-}1x10^8$ (ITu)	5 pts: SD for 7-37 wks
2025[106]	9	Recurrent/ refractory malignant glioma, DIPG, EPN, PB	allo NK cells	$4\text{-}5x10^7$ (IT)	1 pt: PR at 18mth follow-up<br>3 pts: SD at 18mth follow-up

Pt(#): Number of patients, *Diagnosis column:* DIPG: diffuse intrinsic pontine glioma, EPN: ependymoma, GBM: glioblastoma, HER2: human epidermal growth factor receptor 2, MB: medulloblastoma, PB: pineoblastoma*; Treatment column:* allo: allogeneic, auto: autologous, CAR: chimeric antigen receptor, haplo: haploidentical, IFN: interferon, IL: interleukin, NK: natural killer*; Cell dose column:* ICV: intraventricular, IT: intrathecal, ITu: intratumoral, IV: intravenous*; Best clinical outcome column*: MR: minor response, mths: months, PR: partial response, SD: stable disease, wks: weeks.
